# Quantifying the contribution of the rare biosphere to natural disturbances

**DOI:** 10.1093/ismejo/wraf129

**Published:** 2025-06-26

**Authors:** Jianshu Zhao, Genevieve Brandt, Jessica L Gronniger, Zhao Wang, Jiaqian Li, Dana E Hunt, Luis M Rodriguez-R, Janet K Hatt, Konstantinos T Konstantinidis

**Affiliations:** Center for Bioinformatics and Computational Genomics, Georgia Institute of Technology, Atlanta, GA 30332, United States; School of Biological Sciences, Georgia Institute of Technology, Atlanta, GA 30332, United States; School of Biological Sciences, Georgia Institute of Technology, Atlanta, GA 30332, United States; Marine Laboratory, Duke University, Beaufort, NC 28516, United States; Marine Laboratory, Duke University, Beaufort, NC 28516, United States; Marine Laboratory, Duke University, Beaufort, NC 28516, United States; Marine Laboratory, Duke University, Beaufort, NC 28516, United States; Biology Department, Duke University, Durham, NC 28516, United States; School of Civil and Environmental Engineering, Georgia Institute of Technology, Atlanta, GA 30332, United States; Department of Microbiology, University of Innsbruck, Innsbruck, 6020, Austria; Digital Science Center (DiSC), University of Innsbruck, Innsbruck, 6020, Austria; School of Civil and Environmental Engineering, Georgia Institute of Technology, Atlanta, GA 30332, United States; Center for Bioinformatics and Computational Genomics, Georgia Institute of Technology, Atlanta, GA 30332, United States; School of Biological Sciences, Georgia Institute of Technology, Atlanta, GA 30332, United States; School of Civil and Environmental Engineering, Georgia Institute of Technology, Atlanta, GA 30332, United States

**Keywords:** rare biosphere, metagenomics, metagenomics assembled genomes (MAGs), disturbance, resilience, generalists and specialists

## Abstract

Understanding how populations respond to disturbances represents a major goal for microbial ecology. While several hypotheses have been advanced to explain microbial community compositional changes in response to disturbance, appropriate data to test these hypotheses is scarce, due to the challenges in delineating rare vs. abundant taxa and generalists vs. specialists, a prerequisite for testing the theories. Here, we operationally define these two key concepts by employing the patterns of coverage of a (target) genome by a metagenome to identify rare populations, and by borrowing the proportional similarity index from macroecology to identify generalists. We applied these concepts to time-series (field) metagenomes from the Piver’s Island Coastal Observatory to establish that coastal microbial communities are resilient to major perturbations such as tropical cyclones and (uncommon) cold or warm temperature events, in part due to the response of rare populations. Therefore, these results provide support for the insurance hypothesis [i.e. the rare biosphere has the buffering capacity to mitigate the effects of disturbance]. Additionally, generalists appear to contribute proportionally more than specialists to community adaptation to perturbations like warming, supporting the disturbance-specialization hypothesis [i.e. disturbance favors generalists]. Several of these findings were also observed in replicated laboratory mesocosms that aimed to simulate disturbances such as a rain-driven washout of microbial cells and a labile organic matter release from a phytoplankton bloom. Taken together, our results advance understanding of the mechanisms governing microbial population dynamics under changing environmental conditions and have implications for ecosystem modeling.

## Introduction

Natural environmental microbial communities generally harbor a few abundant taxa and numerous low-abundance, or rare, taxa [1]. The rare biosphere is defined as the active or dormant low-abundance taxa in a given environment at a specific time point, typically corresponding to <0.1% relative abundance. Rare taxa are important contributors to both α- and β-diversity, and such taxa have often been shown to have important ecological roles in specific ecosystems [2]. Both the number of taxa (phylogenetic diversity) and the genes these taxa carry (functional diversity) are thought to provide an “insurance” or “buffering/caching” capacity for the ecosystem against environmental change [3, 4]. For example, a rare coastal, oil-degrading bacterial population thrived [i.e. made up ~30% of 16S rRNA gene libraries] three weeks after an oil spill, and then returned to the rare biosphere two months later] once the oil was degraded [5]. Similarly, members of the rare biosphere can also become abundant after shifts in salinity and organic carbon and play important roles in maintaining ecosystem processes [6]. Further, rare taxa can periodically become abundant, and their abundance dynamics may depend on varied selection pressures such as those imposed by seasonal fluctuations in environmental parameters and substrate availability. In addition to these “resource-availability-driven” processes, other ecological and/or stochastic processes including dispersal, predation, (stochastic) reactivation from dormancy, random birth/death, and diversification could also explain the emergence of abundant taxa from rarity [7, 8]. Despite these studies that have documented the role of the rare biosphere qualitatively, an approach to quantify the role of rare taxa more precisely [i.e. how much and how often rare taxa contribute compared to abundant taxa], especially during disturbance events, has been elusive.

To develop an approach that quantifies the role of the rare taxa, it is important to first decide what constitutes a rare taxon. Rare and abundant populations have largely been defined based on arbitrary thresholds of abundance, commonly inferred using 16S rRNA gene amplicon sequence data. Several different relative abundance thresholds have been proposed [e.g. <1%, 0.1%, or 0.01% of the total community], without attention to ecological theory or habitat differences [9–11]. Moreover, defining rare and abundant taxa based on 16S rRNA gene amplicon sequence data can be influenced by biases introduced during amplification and other processes [e.g. abundant populations are favored during amplification and/or sequencing] [12]. More importantly, amplicon sequencing provides little information on population-level functional potential, and thus the functional roles of taxa in maintaining important ecosystem processes. Metagenomics provides the means to capture the functional diversity of the rare biosphere and largely sidesteps the biased amplification of gene-amplicon data [13, 14]. However, it remains challenging to reconstruct the metagenome-assembled genomes (MAGs) of rare taxa due to insufficient sampling (sequence coverage) and to define rare vs. abundant taxa at the genome level [15].

Ecological theories have been developed to explain the responses of abundant and rare microbial populations to disturbance. For example, the insurance hypothesis suggests that the rare biosphere can mediate the effects of environmental disturbances [e.g. oil spill example above], and thus contribute to community resilience [16, 17]. Specifically, this hypothesis predicts that rare species, often below the limit of detection, and thus not included in estimates of community diversity, may quickly respond to altered environmental characteristics (pulse disturbances) and become abundant before returning to pre-perturbation rarity [18]. In contrast, the specialization-disturbance hypothesis suggests that niche breadth, rather than relative abundance, determines how species responds to disturbance. That is, disturbance generally negatively affects specialists, while generalists benefit from disturbance [19]. Defining generalist vs. specialist taxa in environmental samples relies on information about their prevalence or niche breadth: generalists typically show no/small preference for specific environments, contrasting with specialists, which are abundant only in some environments or conditions. Both theoretical and laboratory studies have shown that generalist taxa can be crucial in maintaining ecosystem functioning during disturbance events compared to specialists due to their metabolic flexibility [20, 21]. However, defining generalists and specialists and the effect of disturbance on them have been challenging, due to difficulties associated with defining niche breadth and determining abundance for populations at or near the detection limit.

**Figure 1:**
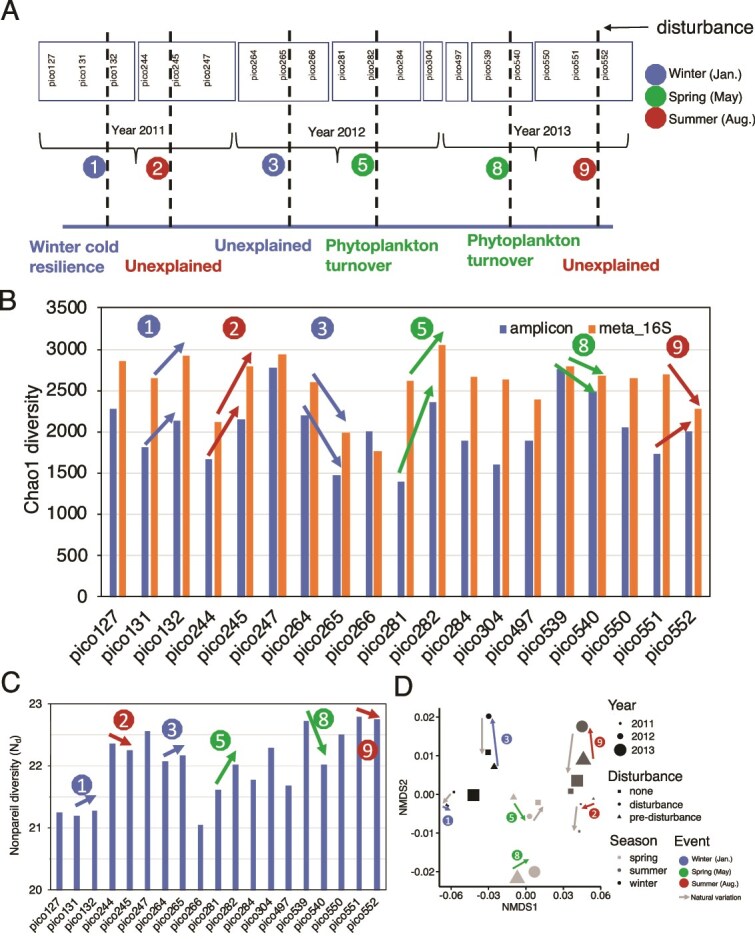
Characteristics of the disturbance events studied here and their effects on microbial community diversity. (A) Details of the PICO time series samples. Sample names are labeled chronologically within boxes, the color of which corresponds to the season along the three-year sampling period (see key). Continuous numbers in the name of samples indicate samples taken one week apart [e.g. pico281 and pioc282 samples are taken 1 week apart, while pico284 is sampled 2 weeks after pico282]. Each vertical line indicates a disturbance event and is labelled with a number for convenience. Description below each vertical line shows the details of each disturbance; additional details are provided in Table S1 and S2. (B) Chao1 diversity index for both amplicon 16S rRNA genes (blue) and extracted 16S rRNA gene reads from metagenomes (orange) are shown [using OTUs defined at 97% identity, see methods; similar patterns were observed with the Shannon index (data not shown)]. (C) Nonpareil diversity (N_d_). (D) Mash distance based NMDS of metagenomes subsampled at the same sequencing effort. Colored arrows in (B), (C) and (D) show how Nd diversity and metagenomic composition changed by each disturbance event. Grey arrows in (D) represent the change in community composition following the disturbance event caused by either natural variation and/or recovery (the beginning of the arrow denotes pre-disturbance and the end of it denotes during disturbance samples). Note that not all events included post-disturbance samples and that some of the compositional shifts observed based on whole-metagenomes may not match perfectly the shifts observed based on 16S rRNA gene amplicon data (Fig. S3), which were used to defined disturbance events (see text for additional discussion). Also, events that affected small parts of the community, and thus did not result in substantial changes in community composition to be discernible by our analysis represented in the graph of panel (D), were not included in our study.

In this study, we sought to quantify how often and by how much rare populations respond to disturbances and what functions these populations provide relative to abundant taxa for coastal microbial communities. In other words, we aimed to directly test the specialization-disturbance and the insurance hypotheses and offer a more quantitative dataset compared to previous (more) qualitative studies of the role of the rare biosphere. A key assumption underlying our quantitative view is that the total cumulative relative abundances of rare vs. abundant taxa are directly proportional to their relative contributions. Further, we defined rare vs. abundant populations based on the species abundance distribution curves and generalists vs. specialists based on a proportional similarity index (PS index). We applied this methodology to time-series metagenomes from surface waters at a coastal observatory (Beaufort, North Carolina, USA) over a three-year period. Disturbances were previously operationally defined based on 16S rRNA gene amplicon data as greater-than-expected changes in community composition and structure relative to seasonally-averaged changes (Fig. 1A, and in [22]). These disturbances often -but not always- represented a major weather event such as a tropical storm or the arrival of a cold front in the area (Table S1). Therefore, we both quantified the importance of the rare biosphere during disturbance and developed a new methodology to define rare vs. abundant taxa at the genome level. We also tested the field data (open system) findings with closed, replicated laboratory mesocosms that aimed to simulate similar natural disturbances.

## Materials and methods

### Sample collection and description of disturbance events

Water samples were collected as part of the Piver’s Island Coastal Observatory (PICO) time series at the mouth of the Newport River Estuary Beaufort, NC, USA (34°71.81’N, 76°67.08’W) on a weekly basis [22]. Although located at the mouth of an estuary, the site is generally representative of the mesotrophic coastal ocean, except for periods of high freshwater flow [23]. Seawater was collected at 10:30 AM  (morning) local time using a 5-liter Niskin bottle centered at 1 m on a peristaltic pump with the tubing open at 1 m and processed within 1 h. Standard laboratory methods for determination of water temperature, pH, salinity, dissolved inorganic nutrient concentrations, and chlorophyll *a* concentrations were described previously [23].

Here, we examine 19 metagenome samples from the period between January 2011 to December 2013, covering the winter, summer, and spring seasons. Samples for metagenome sequencing were selected to capture pre-disturbance, disturbance, and post-disturbance periods. Disturbance events were previously identified as those that changed substantially microbial community composition compared to the weekly expected change based on 16S rRNA gene amplicon sequencing ([22] and Fig. 1A). We also examine the environmental parameters that might have driven these observed community changes to further narrow down the number of events to focus on (Tables S1 and S2). Briefly, for the six disturbance events identified previously by our team [22], disturbance 1 is a presumed resilience event following unseasonably cold water temperatures (denoted as Disturbance_1_Winter_cold_resilience). Disturbance 2 was an unexplained disturbance event (denoted as Disturbance_2_unexplained) and it was characterized by changes in several environmental parameters, [i.e. increases in ammonium (65%), silicate (80.2%), and chlorophyll *a* (78.2%) compared to pre-disturbance conditions]. Disturbance 3 was also an unexplained disturbance (denoted as Disturbance_3_unexplained), characterized by 310% higher ammonium. Disturbance 5 occurred in the spring of 2012 and was a presumed phytoplankton turnover event (denoted as Disturbance_5_Phytoplankton_turnover); consistently, we observed 116% higher NOx, 55.9% lower silicates, and 31.7% lower chlorophyll *a*. Disturbance 8, occurred at roughly the same time of year as Disturbance 5 and was also labeled a spring turnover event (denoted as Disturbance_8_Spring_turnover); it was characterized by 100.1% more chlorophyll *a* and 59% lower ammonium. Both Disturbances 5 and 8 were defined as spring turnover events based on changes in their phytoplankton community composition rather than environmental parameters [22]. Finally, Disturbance 9 was an unexplained event (denoted as Disturbance_9_unexplained) and characterized by higher ammonium (220.3%), silicate (49.6%), chlorophyll *a* (20.2%), and temperature (1.5°C see also Supplementary Tables S1 and S2 for more details about each event). Depending on the disturbance event, environmental parameters returned (resilience events) or did not return (non-resilience events) to the seasonal (typical) values post-disturbance (Table S1). Specifically, silicate (SiO_4_-) and chlorophyll *a* concentration recovered one week after disturbance events 5 and 9. In contrast, for event 2, we only saw recovery of NH_4_^+^ and oxygen availability, and other factors did not return to pre-disturbance values.

### Mesocosm experiment design, setup, and amplicon sequence analysis

To further investigate microbial community responses to major storm associated events (freshwater discharge and phytoplankton blooms) under controlled conditions, we performed laboratory-scale manipulation experiments. We designed these experiments to test the impact of two key processes on microbial community composition: (i) a dilution of the microbial community from the influx of freshwater that reduces competition without adding new resources, and (ii) a phytoplankton-bloom that reflects phytoplankton blooms, which commonly follow a major influx of freshwater caused by a storm. The experiment was conducted in an environmental chamber that aimed to simulate *in situ* light and temperature conditions. Experimental manipulations involved triplicate carboys assigned to either control, a 1:10 dilution of the bacterioplankton community using 0.2 μm filtered PICO seawater (no change in salinity), or a daily treatment of dissolved organic matter (DOM) through the addition of lysate of the bloom-forming diatom *Skeletonema costatum*. This addition represents a projected ~45% increase in daily primary production relative to non-hurricane conditions, which was achieved by adding ~2 ml of concentrated lysate daily to increase the whole mesocosm’s carbon levels by 10 μM. The mesocosms were incubated for 5 days before being sacrificed and samples obtained for metagenome sequencing. See Supplementary Material for additional details about experimental procedures, including detailed mesocosm description, DNA extraction, and sequencing.

### Metagenomic sequencing, quality control, and coverage estimation

For metagenomes, 10 ng of DNA was sheared using the Covaris LE220 and 300bp sized fragments were selected using SPRI beads (Beckman Coulter). The fragments were treated with end-repair, A-tailing, and ligation of Illumina compatible adapters using the KAPA-Illumina library creation kit followed by five cycles of PCR to enrich for the final library. These libraries were sequenced with a 2 × 150 nt read strategy on the HiSeq 2500 1 T System (Illumina) platform at either the Joint Genome Institute or the Duke’s Genome Sequencing and Analysis Center. Adapter trimming and demultiplexing of sequenced samples was carried out by the instrument. Raw reads were trimmed using Trimmomatic with default parameters [24] and then checked using FastQC (https://github.com/s-andrews/FastQC). Sequence subsampling to account for sequencing depth variation among libraries was done using Seqtk and specifically, the subseq command with the same random seed for forward and reverse reads (https://github.com/lh3/seqtk). Sequencing coverage estimation and metagenome alpha diversity were calculated using Nonpareil v3.0 with -kmer option [25].

### 16S rRNA gene-carrying read extraction from metagenomes, closed reference OTU picking, and 16S rRNA gene amplicon sequence analysis

Prokaryotic 16S rRNA gene-carrying metagenomic reads were extracted using metaxa2 [26]. USEARCH closed_ref workflow in USEARCH v11.0.667 was performed to pick closed reference OTUs (not ASVs since 16S rRNA gene short sequences extracted from metagenomes are usually not alignable) from the extracted 16S rRNA gene-carrying short reads with default identity of 97% (a 98.5% identity threshold did not differentiate our conclusions substantially) [27, 28]. Briefly, USEARCH was used to align extracted short reads against the non-redundant SILVA database v138.1. If the semi-global alignment identity of a query sequence to a database sequence was better than 97%, the input sequence was assigned to that OTU. Otherwise, the input sequence was discarded and not assigned to an OTU (an identity threshold of 99% was also used but only small differences were observed in the results in terms of community composition, especially at higher taxonomic levels than the family level). When multiple identical matches to different reference sequences above the threshold were obtained, we randomly kept one of the matches. Extracted 16S rRNA gene reads were rarefied to 8000 sequences/sample before downstream analysis (Fig. S2). Note that closed reference OTU clustering may not find an OTU for a (query) sequence that is not represented with a closely related sequence in the SILVA database, contrasting with de novo OTU/ASV clustering using amplicons. However, such sequences were rather rare in our datasets, representing <5% of the total sequences for each sample; hence, discarding them from further analysis should not affect downstream results and conclusions. Downstream analyses including diversity and composition analysis were performed using MacQIIME v1.9.1. See Supplementary Material for details of 16S rRNA gene amplicon sequencing and analysis.

### Metagenome assembly, contig taxonomic classification, and functional gene annotation and diversity analysis

Quality filtered short reads were assembled with Megahit v2.1.2 (parameters: --meta --min_contig 1000) [29]. To classify the assembled contigs taxonomically, the Centrifuge program was used to search against the RefSeq complete genome collection with default parameters [30]. Genes were predicted on contigs using Prodigal v2.6.3 (−p meta) [31]. After mapping short reads onto each contig and the genes of a contig using bwa-mem2 [32], contig/gene coverage (or abundance) was calculated by the CoverM v0.6.1 (https://github.com/wwood/CoverM), contig workflow with abundance metric option metabat (--methods metabat --min-read-percent-identity 0.95 --min-read-aligned-percent 0.75). Diamond v0.9.22 was used to perform functional annotation of predicted genes against the Swiss-Prot database (Diamond blastp -k 1 --id 40 --query-cover 70 --max-hsps 4 -e 0.0001) [33]. The matching Swiss-Prot reference sequences were mapped to the Gene Ontology (GO) terms and filtered for molecular functions. The Chao-Shen estimate of Shannon entropy H for molecular functions and cellular processes was calculated using the R package Entropy based on the observed read counts (coverage) of genes assigned to the same molecular function GO term. This estimation adjusts for missing species (here GO terms) and sample coverage. The exponential of the estimated Shannon entropy was used to covert the statistic to true diversity (^1^*D*) with units of effective GO terms as described recently [34]. The fraction of the total proteome devoted to extracellular proteins for each MAG was predicted using psortb [35].

### Population genome binning and bin/MAG refinement

Maxbin 2, Metabat2, and CONCOCT were individually applied to contig binning with default parameters [36–38]. The resulting MAGs were first refined (quality improvement) using DAS_Tools with the searching engine USEARCH [28, 39]. Refined MAGs were further checked by CheckM unique command to ensure that no contigs were binned to the same MAG more than once and one contig was not binned to multiple MAGs. Mis-binned contigs were removed from MAGs. Contig coverage depth and tetranucleotide frequency of each MAG were subsequently evaluated to determine whether binned MAGs were likely to be chimeric sequences using an in-house R script (available at https://github.com/jianshu93/bin_check). CheckM lineage_wf was then used for MAG quality assessment with default parameters [40]. The quality score was defined as completeness - 5^*^contamination + Strain heterogeneity^*^0.5. Medium to high quality MAGs were defined as quality score larger than 0.5. To further check whether the MAGs binned from each sample represent sequence-discrete populations (species) [41], competitive read mapping on the MAGs followed by recruitment plot were performed via the scripts in the enveomics R package [42] (the implementation used is available at: https://github.com/jianshu93/RecruitmentPlot_blast). Briefly, contig sequences of all MAGs from the same sample were labeled and then pooled together as one genome database. Subsequently, a blastn search was performed to map quality-controlled short reads to the contigs (−task blastn -id 95% -max_target_seqs 500). The blastn tabular output was filtered to remove mapped reads with a low alignment ratio (<90% of read length) and to keep only the best match for each query read according to identity. Tied matches were also removed before creating a recruitment plot using the BlastTab.recplots2.R. MiGA quality_wf with the MyTaxa option was then performed to further validate the taxonomic identity of the contigs binned into MAGs [43] and also calculate gene coding density, %G + C content, and other descriptive statistics such as contig length [44].

### MAG dereplication and taxonomic classification

Dereplication of the MAGs was performed using dRep v2.2.4 with an ANI threshold of 95% by the -fastANI option (minimum completeness 70%, maximum contamination 10% and quality score > 0.5 for filtering MAGs before dereplication) [45]. Quality information from CheckM was passed to dRep to select the best quality MAG as representative of each resulting 95% ANI cluster. MAGs were classified against the Genome Taxonomy Database v214 [46] using the GTDB-Tk classify_wf workflow [47]. To further confirm the taxonomy assigned to each MAG by GTDB-Tk, especially those that were not classified with confidence [e.g. distantly related to their best matches found in GTDB], we used the MiGA workflow miga classify_wf against the type material database [44]. The lowest taxonomic level to which MiGA considered the assignment of the query MAG as significant was kept and compared to the GTDB-Tk classification results.

### Relative abundance calculation and functional gene annotation of MAGs

The relative abundance of each dereplicated MAG was calculated by competitively mapping reads from each sample to the entire dereplicated MAG collection using bowtie2, followed by SAMTools to generate sorted BAM files for each MAG [48, 49]. Bam files were subsequently filtered using the CoverM workflow (--min-read-percent-identity 0.95 --min-read-aligned-percent 0.75) and only reads with an alignment ratio and identity larger than 75% and 95%, respectively, were kept. Truncated Mean Depth 80% (TAD80) was then calculated as a proxy for relative DNA abundance, which normalizes for highly conserved or variable regions of the genome [50]. TAD80 estimates were further normalized by genome equivalents based on MicrobeCensus [51] to account for average genome size differences between the samples and provide the final (normalized) relative abundance estimates, using an in-house script (https://github.com/jianshu93/Competitive_mapping) [50]. Functional annotation of the dereplicated MAGs were performed using MicrobeAnnotator and DRAM [52, 53]. Briefly, MicrobeAnnotator searches multiple reference protein databases iteratively and combines results from KEGG Orthology, Enzyme Commission, GO, Pfam and InterPro, and returns the matching annotations together with key metadata. DRAM profiles microbial (meta)genomes for metabolic processes like carbon degradation, photosynthesis, methanogenesis, known to impact ecosystem function across biomes.

### Proportional similarity index for defining generalist and specialist MAGs

Levins’ niche breadth index (average relative abundance of species in different environments) has recently been used to define generalists vs. specialists for microbial populations [50, 54, 55]. However, this metric assumes equal availability of resources, which is rarely the case in natural environments. A metric based on the proportional similarity index, which defines generalists and specialists independently of their absolute abundance and occupancy, was used here to circumvent this limitation as suggested previously [56, 57]. Specifically, habitat breadth was defined by the proportional similarity (PS) index [56] relating the proportion of a population found in each category of samples to the proportion of sampling effort (total samples) for that category as follows: $P{S}_{index}=1-0.5\sum_i\mid{p}_i-{q}_i\mid$, where p_i_ is the number of cells [we use relative abundance, i.e. TAD/genome equivalent] of the target species in samples of category i [i.e. season], divided by the total number of cells from that species in all available samples, while q_i_ is the number of all the cells in samples of category i, divided by all the cells in all samples. Thus, PS index values express variation in the habitat breadth of a species, which is an important aspect of the realized niche of the species (preferences for samples/environments), and range between 0 and 1 for the broadest possible and the narrowest possible niche, respectively [i.e. a population is restricted exclusively to the rarest category of sample types and consequently, is absent in all other types]. MAGs detected in only one metagenome sample were classified as rare species and excluded from the habitat breadth [i.e. generalist vs. specialist] analysis as no reliable measure of niche breadth could be calculated for such populations. For our time series dataset, samples were first grouped into three seasonal types (or environments, see Table S1 for details) and then sub-typed within each main type by year that the samples were obtained (Table S4). Seasons were defined based on the yearly calendar, but this was also consistent with distinct environmental conditions between seasons at our sampling site (see Table S1 for details). The PS index was calculated separately for each category of samples (the three main types and the eight sub-types), generating two scores (PS index_subtype_ & PS index_maintype_). Species that scored in the upper third percentile for both categories, indicating a broad niche, were classified as habitat generalists (each species was represented by an individual MAGs after dereplication in our analysis). Species (or MAGs) that scored in the bottom third percentile for both categories were classified as habitat specialists, and were further subdivided into regular habitat specialists (rank-transformed; i.e. the rank of the PS values, PS index_subtype_ > PS index_maintype_) and strict habitat specialists (rank-transformed; PS index_subtype_ < PS index_maintype_) [57] (Table S4). This definition allows species that are abundant in some samples and widespread (but not equally abundant in all samples) to be classified as habitat specialists when high abundances are observed in a single category of samples [e.g. single season] but not in all categories of samples [e.g. abundances are much lower in the latter] [57]. We also compared this PS index with Levins’ Breadth Index and the PS index implementation in MicroNiche package, which provides null model tests with respect to the limit of detection [58]. Specifically, for the PS index calculated by MicroNiche, we used the principal component 1 of all available resource variables [e.g. DIC, chlorophyll *a*, NH_4_^+^, NO_3_^+^ NO_2_, PO_4_^3−^, SiO_4_^4−^] as the resource parameter.

### Diversity analysis and statistics

All diversity and statistical analysis were performed using the R language (version 4.0.5) and the vegan and stats packages. The ggplot2 package was used for figures.

## Results

### Changes in overall microbial community composition in response to disturbance events

As our metagenomes were not sequenced with the same effort and in order to avoid any effects of metagenome size on the conclusions [e.g. the species abundance curve to define rare taxa], we first subsampled the metagenomes to the level of the smallest metagenome. The analyses below are based on the subsampled metagenomes unless otherwise noted. Shifts in microbial community diversity did not show a consistent pattern between disturbance events. The Chao1 index based on either 16S rRNA gene fragments recovered from metagenomes or 16S rRNA gene amplicon sequences (using OTUs defined at 97% identity; see Methods) neither increased nor decreased systematically for all events (Fig. 1B). Similarly, Nonpareil diversity, a reference database-free metric combining richness and evenness based on shotgun metagenomic data, did not identify a consistent alpha diversity response to disturbance (Fig. 1C and Fig. S1). However, no matter whether disturbances increased or decreased diversity, diversity largely recovered after each disturbance, indicating microbial community resilience. This community resilience following disturbance was also supported by beta diversity analyzed based on a NMDS of Mash distances of whole metagenomes, or amplicon and extracted metagenomic reads carrying 16S rRNA gene fragments (community composition level; Fig. 1D and Fig. S3). Functional diversity analysis showed limited change with disturbance despite substantial diversity and taxonomic composition changes (Fig. S5), indicating high functional redundancy among the sampled microbial communities, consistent with previous large scale studies of microbial communities [59].

### Identifying abundant vs rare microbial populations

For time series samples, a total of 394 medium or high-quality MAGs [i.e. quality score > 0.5] were obtained after quality control and before de-replication at 95% ANI. For each sample, contigs of different MAGs fell into separate clusters based on dimension reduction of contig information [i.e. contig coverage and kmer profile] using the mmgenome2 software (Fig. S11), confirming that most MAGs were likely not chimeric and represent species-level clusters (or genomospecies). Recruitment plots showed that each MAG represented a sequence discrete population within the sample from which it was recovered [i.e. reads mapping between 85%–95% nucleotide identity were rare compared with reads showing >95% identity to the MAG (Fig. S12)]. These results were consistent with previous findings suggesting that prokaryotic species are distinguishable in natural communities [41]. Among the 198 non-redundant MAGs after dereplication, three archaeal MAGs were found, belonging to the recently proposed family *Candidatus* Poseidonaceae (formerly subgroup MGIIa) and one MAG belonging to *Ca*. Thalassarchaeaceae (formerly subgroup MGIIb). Among the bacterial populations, although the order *Pelagibacterales* was very abundant at the read (9.6% to 22.5% of the total metagenome/community) or assembled contig levels (4.4% to 5.2% of total contigs), only one *Pelagibacterales* MAG was recovered with medium quality (quality score 0.57). All other *Pelagibacterales* MAGs were low in completeness and were removed at the MAG quality control check step. Inability of current genome binning algorithms to handle high intra-population diversity and recover representative genomes has been previously noted for this bacterial group [60]. Most other MAGs were assigned to the classes of *Flavobacteriia* (phylum *Bacteroidetes*; recently renamed to *Bacteroidota* (n = *21*), *Alphaproteobacteria,* and *Gammaproteobacteria* (phylum *Proteobacteria/Pseudomonadota (n = 33 and 17, respectively*), with a few MAGs assigned to other phyla (Table S3). These dereplicated MAGs collectively represented 5.1% to 18.2% of the total metagenomic reads, depending on the sample considered, consistent with the high overall diversity of our samples.

**Figure 2:**
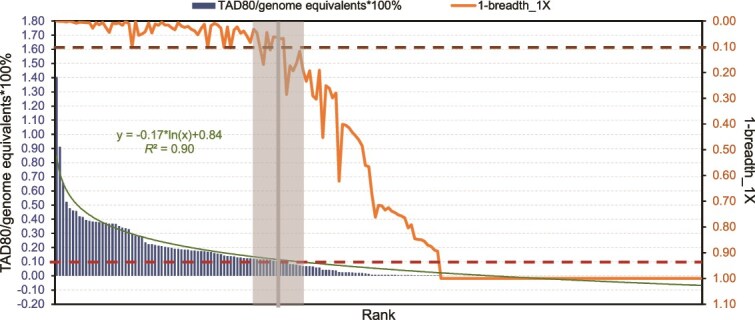
Graphical representation of our approach to define abundant vs. rare MAGs. MAG coverage depth (left y axis, blue bars) and coverage breadth (right y axis, orange line, shown as 1- coverage breadth) distribution for one metagenomic sample (pico127). The X axis is MAG rank by abundance, estimated as coverage depth, [i.e. TAD80 values normalized by genome equivalents]. Dashed blue and orange lines represent the normalized coverage depth of 0.1% and the coverage breadth of 0.1, respectively. The shaded grey region and vertical line (center of area on the x axis) indicate where both coverage depth and breadth drop sharply as abundance rank increases. The vertical line was therefore used to delineate abundant (left of the line) vs. rare (right of the line) MAGs. The green line is a log fitting of coverage depth vs. rank abundance with the function shown above the line. For detailed model fitting of coverage depth distribution, see Supplementary Fig. S6.

We competitively mapped reads from each metagenome to the dereplicated MAGs and built species abundance curves for each genomospecies represented by a MAG (Fig. 2). We used the resulting species abundance curve to examine if there is any natural discontinuity or inflection point that could be used to define abundant vs. rare taxa more reliably than previous arbitrary abundance thresholds (discussed above). The species abundance curve was modeled robustly by a log scale model (Fig. 2, Fig. S7 (a) and (b), *R*^2^ = 0.73 ~ 0.90, *P* < 0.01), and we observed no region of discontinuity in the data. However, a sharp decrease in the slope of the curve was observed, around 0.1% relative abundance in most of the samples (Fig. S6). We also observed a corresponding decrease in MAG breadth coverage (how much of the length of the genome is covered by reads) for relative abundance around 0.1% (Figs. 2 and S7). Specifically, breadth of coverage was 90% or more at this level of relative abundance and decreased quickly for less abundant MAGs. Based on these results, we defined MAGs as rare when they showed relative abundance less than 0.1% and a coverage breadth less than 90% in that sample; we adjusted this threshold on a per-sample basis (ranging from 0.08 to 0.2%), depending on the sample’s species abundance curve [i.e. a corresponding decrease in abundance and breadth coverage]. In contrast, abundant MAGs were defined as those showing greater than 0.1% relative abundance and coverage breadth more than 90%. Robust detection of a MAG and estimation of its relative abundance is achieved with breadth 10% or higher [61]. Hence, MAGs are reliably detectable at 0.1% relative abundance or somewhat lower, and the 0.1% abundance threshold is not a mere artifact of sequencing effort applied [e.g. inability to detect a MAG], and largely agrees with the literature on the rare biosphere [62].

### Evaluating the insurance hypothesis: rare MAGs often contribute to community response

**Figure 3:**
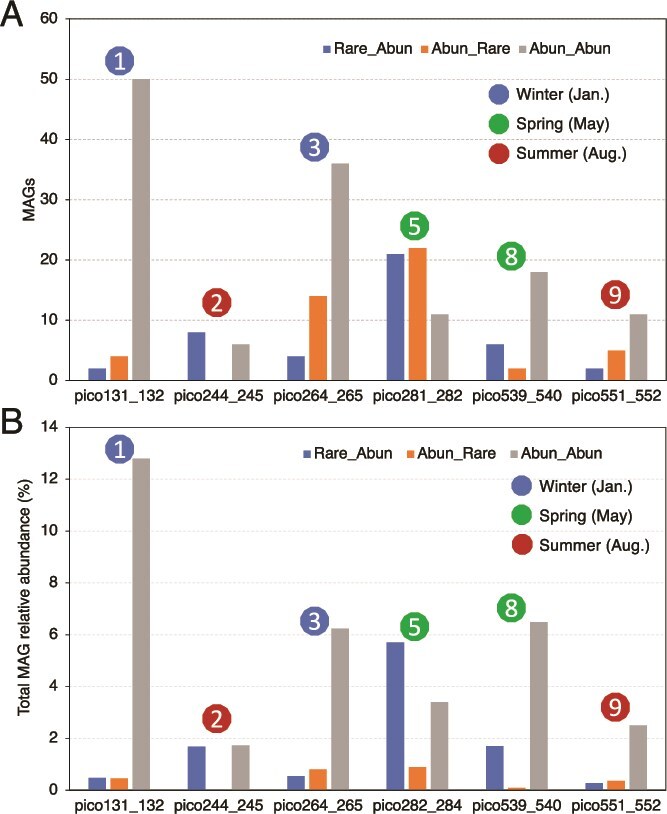
Response of abundant vs. rare MAGs to disturbance events. (A) The figure shows the number of MAGs for each of the three categories assessed: abundant MAGs that remained abundant after the event, MAGs transitioning to abundant from rare, and MAGs becoming rare from abundant for each of the disturbance events. For one given event, if MAG’s relative abundance and coverage breath fall below the threshold of being abundant in the pre-disturbance sample and fall above the threshold of abundant in the disturbed sample, this MAG will be in the category of Rare_Abun. Similar rules applied for other two categories based on MAG abundances before and after each disturbance event. Disturbance events are numbered as in Fig. 1. (B) Total cumulative relative abundance of MAGs assigned to each category.

We first tested for evidence in support of the insurance hypothesis in each of the six disturbance events sampled by our time series metagenomes. Since our metagenomic sampling is part of a much longer (3-year) 16S rRNA gene amplicon-based time series dataset, we selected samples for metagenome sequencing to represent the pre-disturbance, disturbance, and post-disturbance conditions based on the 16S rRNA gene compositions. That is, pre-disturbance samples were within seasonal norms, and thus served as useful controls (reference points) for assessing disturbance effects [63]. We first assessed the overall MAG relative abundance changes across all disturbance events and found that for each event, many MAGs showed clear changes when comparing disturbed samples with pre-disturbed samples (Fig. S8). We found that for all six disturbance events, between 2 and 22 genomospecies represented by MAGs (Fig. 3A) switched from rare to abundant using the criteria established above. Further, these MAGs made up between 0.5% to 6.9% of the total community based on mapped reads (Fig. 3B). There were also eight to 29 MAGs that switched from being abundant to rare, representing 0.4% to 1.8% of total reads in the disturbed sample. For each event examined, more MAGs transitioned from abundant-to-rare than the reverse, except during the disturbance event 8 (Fig. 3A). Further, the fraction of reads mapping to rare-to-abundant MAGs was higher than that for abundant-to-rare MAGs in the disturbed samples in three out of the total six events (Fig. 3B). It should be mentioned that 5.1% to 18.2% of the total reads in a sample were mapped to all available MAGs (including MAGs in the three categories above and also those in the rare-to-rare category), which represents a substantial fraction of the microbial communities sampled that is similar, if not larger, than the fraction assessed by most previous studies of the rare biosphere based on isolates or laboratory incubations. Collectively, these results revealed that rare species contributed substantially to the community’s response to disturbance, making up about one third of the total abundant taxa during disturbance for all events (based on mapped reads), providing support for the insurance hypothesis.

Although we can only make limited ecological inferences from our data due to the lack of independent replicated disturbance events (no biological replicates available), we can draw on specific instances to develop a better understanding of microbial community response to disturbance. As an example, for disturbance #5 (Disturbance_5_Phytoplankton_turnover) representing a spring phytoplankton turnover event, 21 rare MAGs became abundant (7% of total community), while 29 abundant MAGs switched to rare (initially 5.3% but 1% of the total community following the disturbance; Fig. 3b). Sixteen MAGs remained abundant during this event, representing 4.5% of the total community. Further analysis showed that the rare MAGs that became abundant during this spring turnover event encode more metabolic pathways related to carbohydrate degradation and fewer pathways related to photosynthesis compared to abundant-to-rare MAGs [i.e. abundant MAGs represented a shift from photoautotrophic *Synechococcus* spp*.* to heterotrophs; Fig. S13]. These results suggested more carbon compounds were available in the surface seawater compared to pre-disturbance, and genomospecies that were more efficient in utilizing those compounds gained a competitive advantage over those that lacked these pathways [e.g. abundant to rare MAGs]. Amplicon-based analysis also showed consistent results: shifting from *Synechococcus* spp*.* to diatoms within the phytoplankton [22]. These results suggest that rare populations provided insurance for ecosystem functioning during a short-term disturbance. That said, the importance of abundant populations that remained abundant cannot be overemphasized because these MAGs represented a higher fraction of the total community than the rare-to-abundant MAGs in all events, including this disturbance event 5 (Fig. 3B).

There were three disturbance events with post-disturbance metagenomes that allowed us to assess potential recovery of the genomospecies that changed in abundance (Disturbance_2_Unexplained, Disturbance_3_Unexplained Disturbance_5_Phytoplankton_turnover). For Disturbance_3_Unexplained, a relatively small number of MAGs changed category [i.e. transitioned from rare-to-abundant or abundant-to-rare; 25% and 33.3% of the total MAGs in each category, respectively], compared to the disturbance event 5. For Disturbance_2_Unexplained, 44.4% of MAGs transitioned from rare-to-abundant and 25% of MAGs transitioned from abundant-to-rare, and all these MAGs recovered to their pre-disturbance level after the event. For the remaining disturbance events, we observed that two of four MAGs and three of seven MAGs in the rare-to-abundant and abundant-to-rare categories, respectively, recovered to pre-disturbance levels for disturbance 1, while one of two MAGs and two of eight MAGs of the same categories recovered to post-disturbance levels for disturbance 9. Overall, except for disturbance 3, roughly half or more of the rare-to-abundant populations recovered after the event (i.e. returned to rarity), further indicating that while rare populations play a role in community resilience by responding to niches created by the disturbance, most of these taxa return to rarity post-disturbance. However, except for disturbance 5 for which 66.7% of the abundant-to-rare MAGs became abundant again, less than half of the abundant-to-rare populations recovered abundance, indicating that rare and abundant populations may respond differently to distinct disturbances. Disturbance 5 is thought to be a spring phytoplankton overturn event, and contained a large number of taxa whose abundance post-disturbance did not return to their pre-disturbance levels (as shown using the entire 16S rRNA gene dataset) [22], highlighting differences between disturbance types.

### Results from closed manipulation experiments that simulated disturbance events

Our sampling site is a natural ecosystem (open system) that experiences multiple changes in environmental conditions and inputs over short periods of time, likely shorter than our weekly sampling scheme. Further, we did not have replicated disturbances to statistically assess how reliable our results are with respect to which MAGs were identified as changing between abundance categories in response to the disturbances. Such replicated field samples are -in general- technically problematic because the same (natural) disturbance event cannot be replicated and samples taken from the same disturbance on the same or adjacent days are not independent from each other [64]. Therefore, it is likely that several of the MAGs identified as changing between abundance categories represent spurious findings resulting from stochasticity in field sampling and/or sample processing for sequencing. However, we expect that the fraction of the total MAGs that changed with disturbance and their ecological strategies [i.e. generalists vs. specialists; see next section] to be generally reliable.

To test the broader applicability of the field results, we examined 20 L laboratory mesocosms that aimed to simulate disturbance events; specifically, a large rainfall that caused a 10-fold dilution of the bacterioplankton and a phytoplankton bloom that introduced labile DOC (see Materials and Methods for details). The MAGs recovered from these mesocosm metagenomes, using the same methodology as with the PICO field metagenomes, cumulatively made up ~17% of the total community, on average. In the dilution microcosm, 54/322 of the MAGs (total relative abundance 9.06%) transitioned from rare to abundant relative to controls, while 41/322 became rare from abundant (making up 1.48% of total community) and 11/322 remained abundant (7.94% of total community) (Supp. Data S1). For the DOM addition treatment, 24/322 switched from rare to abundant (3.1% of total community) while 8/322 transitioned from abundant to rare (0.6% of total community), while 44/322 MAGs remained abundant (13.17% of total community) (Supp. Data S2). Overall, the results from these mesocosms, in terms of the relative contribution of rare taxa transitioning to abundant, were strikingly similar to those reported above for the field metagenomes. However, we cannot assess resilience in these short-term experiments [i.e. whether MAG abundances returned to pre-disturbance levels] because such mesocosms typically evolve to a new state due to “bottle effects”. Further, when we applied the DESeq2 approach [65] to identify MAGs that changed in abundance between control (n = 3 replicates) and treatment (n = 3 replicates; Fig. S16), the identified MAGs were highly overlapping with those identified as changing between the rare vs. abundant categories based on our simple comparison of mean abundances above the sample-specific abundance threshold (Fig. 2), showing that the latter approach is reliable. We observed consistent results when we compared the MAGs that changed in abundance categories during event #5, which represented a diatom bloom event due to increased spring temperatures, to the mesocosm MAGs of the DOM addition treatment, which aimed to simulate a similar disturbance. For instance, we found that two field MAGs out of five total (~40%), which became abundant from rare and we also recovered a same-species MAG from the mesocosm metagenomes (>98% ANI), showed the same abundance pattern [i.e. became abundant from rare] between the treatment and the control mesocosm incubations. The remaining three MAGs did not change in abundance categories based on the mesocosm data [i.e. remained rare], which could reflect the unique conditions of the mesocosms relative to the field or stochastic (spurious) results as we hypothesized above (Fig. S17). Overall, our results with the replicated mesocosms suggest that most of the findings with the field data are reliable although some stochastic findings, especially on which specific MAGs change between abundance categories during disturbance, are likely present among our field findings.

### Testing the disturbance-specialization hypothesis: generalists are favored by disturbance

**Figure 4:**
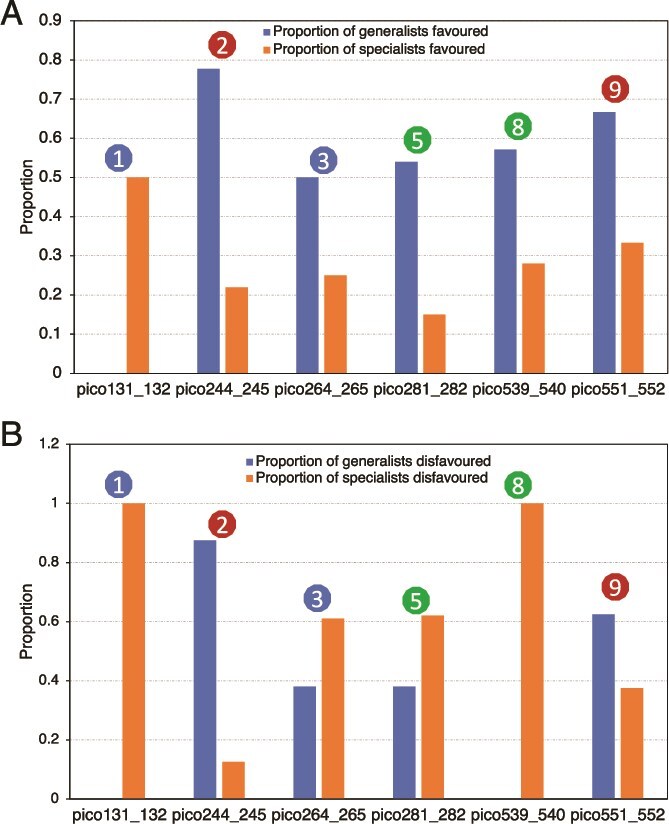
Fraction of specialists vs. generalists selected by each disturbance event. (A) The graph shows the number of MAGs that became abundant from rare [i.e. favored by each disturbance event]. (B) Fraction of specialists and generalists disfavored by each disturbance event. Disfavored MAGs are those that become rare from abundant [i.e. similar to (A) but the opposite pattern]. Disturbance events are numbered as in Fig. 1A.

We examined disturbance-responsive MAGs in terms of generalist vs. specialist lifestyles. Here, we used the proportional similarity index (PS index) that measures the population environmental preference by examining abundance changes across categories of available samples (Table S4). We categorized the upper and lower third of MAGs based on a ranked PS index (see Materials and Methods for details) as generalists and specialists, respectively. Notably, our rank-based definition of generalists is generally consistent with the Levins’ breadth niche index and the PS index implementation in MicroNiche (Fig. S18), but it is more appropriate for our data as detailed in the Methods section. For rare populations that became abundant after the disturbance, a larger fraction of them were generalists (60% to 78%) than specialists (16% to 24%) except for the first winter disturbance, which was a resilience event (Fig. 4A). In contrast, most of the abundant populations that became rare were specialists, except for disturbances 2 and 9 (Fig. 4B); the disturbance 2 metagenome contained an enrichment in phage genes (22). Functional gene content analysis revealed that generalists had smaller genome sizes and more compact genomes than specialists (Fig. S9), and a slightly higher fraction of extracellular function proteins encoded in their genomes (Fig. S10), which echoes our previous results from a large-scale survey of freshwater and brackish metagenomes [50]. These results support the disturbance-specialization hypothesis that disturbances overall favor generalists and explain, at least partially, responses to nearly all disturbance events.

For winter Disturbance_1_Winter_cold_resilience and Disturbance_3_unexplained events, the populations that remained abundant were identified mostly as specialists with only a small fraction of generalists. In contrast, for summer disturbances (disturbances 1 and 9), most MAGs that remained abundant were generalists (6 and 9, respectively), suggesting that these generalists persisted and were not altered by the summer disturbance events. For Disturbance_5_Phytoplankton_turnover, a majority of abundant MAGs that remained abundant before and during the event were identified as specialists (7 of 11), contrasting with Disturbance_8_Spring_turnover, in which a majority of abundant MAGs that remained abundant before and during the event were generalists (17 of 21). These patterns suggested that abundant populations of the community, which could be either generalists or specialists, are generally resistant to the effects of the disturbance events studied, consistent with the results reported above at the whole-community and MAG levels.

## Discussion

Quantifying microbial community response to disturbance represents a cornerstone of microbial ecology and is key to testing ecological theory as well as for modeling global change on microbiomes. We observed that a large fraction of MAGs remained abundant before, during, and after disturbance (resistant) for all the disturbance events studied (Fig. 3A and B), suggesting that these populations are insensitive to short-term disturbances and provide stability for the ecosystem. Additionally, rare populations transitioned to abundant after the disturbance event (0.3% and 7% of the total community based on reads). However, more than half of these populations returned to rarity one or two weeks after disturbance, especially for disturbance events 2, 5, and 9, consistent with the concept of conditionally rare taxa (CRT) defined by Shade and colleagues [7] and also in agreement with the results of amplicon based analysis of the same samples [22]. CRT may therefore provide resilience to a disturbed ecosystem [3], and our approach here quantified this contribution coupled with genomic insights [e.g. gene functions enriched by disturbance]. For most of the events with appropriate post-disturbance samples available to assess recovery, both diversity (Fig. 1A and Fig. S3a and b) and composition (Fig. S4) recovered after disturbance at the whole-community level, in agreement with previous findings in a lake ecosystem [66]. We also found that rare-to-abundant genomospecies harbored more metabolic pathways related to carbohydrate degradation compared to abundant-to-rare genomospecies during a spring disturbance event (Disturbance_5_Phytoplankton_turnover), thus providing a possible function-based explanation for these results.

The enrichment of rare-to-abundant genomospecies in metabolic pathways relative to abundant-to-rare genomospecies was clearly observed only in one of the two spring phytoplankton turnover events (Disturbance_5_Phytoplankton_turnover and Disturbance_8_Spring_turnover), potentially due to differences in the timing of the sampling [e.g. Disturbance_8_Spring_turnover had twice as much chlorophyll *a* (Table S2)]. The lack of universal patterns observed across events is likely due, at least in part, to the distinct background conditions and drivers underlying each disturbance, which would favor different MAGs and functional traits [e.g. high inter-event diversity]. Further, the lack of recovery to pre-disturbance conditions observed for several events could be due to a number of factors including interannual variation in environmental conditions [67] or non-deterministic responses to disturbance and recovery (discussed also below). Additionally, the inconsistent response to different events also reflects -to some extent- our inability to capture disturbance drivers. For example, some genomospecies may take longer (>1 week) to recover to pre-disturbance levels than the timeframe captured by our sampling scheme (weekly samples). Although replicating disturbances is not possible [i.e. each storm is unique], biological replicate samples are possible, and the results obtained here suggest that more replicated samples, especially representing the prevailing, non-disturbance-associated, microbial communities in each season, could be helpful in future time series studies.

We believe that the effect of stochastic processes [i.e. random death/birth] on our conclusions mentioned above is limited compared to that of event-driven deterministic processes. For disturbance events where the post-disturbance samples closely resembled the pre-disturbance 16S rRNA gene-based microbial community composition, such as event 3 (see Fig. S3), the MAG abundances were much more similar between the pre- and post- disturbance samples relative to the disturbance sample (Fig. S15). Therefore, most of the MAGs reported to change between the abundant and rare categories are presumably due to the effects of the events rather than stochastic processes.

How to define rare populations or species is a challenging task, and arbitrary thresholds based on relative abundance of 16S rRNA gene sequences [e.g. 0.1%] have commonly been used [9]. Here, we defined rare populations based on the species abundance curve that was derived from the sequencing depth and breadth coverage of recovered MAGs (normalized by total genome equivalents), while considering the limit of detection of our metagenomic sequencing effort. Specifically, the TAD80 metric used for calculating abundance was larger than the genome breadth coverage that corresponded to the limit of detection, which was defined as -ln(0.9)/genome equivalents [68]. Therefore, the commonly used 0.1% relative abundance threshold for rare taxa identified by our analysis, and our definition of rare MAGs, were robust. Although our derived threshold often matched the previous (arbitrary) thresholds, we anticipate that the threshold may differ for other samples and/or environments due to distinct species abundance curves. Hence, the approach outlined here based on the species abundance curve should be useful for future studies. Further, the TAD80 (coverage depth), a metric used to define MAG abundance, is robust to overestimations [e.g. highly conserved regions and regions recently subjected to horizontal gene transfer are effectively masked] and underestimations [e.g. caused by highly variable gene-content regions of the genome]. Thus, the TAD80 provides reliable estimations of the relative abundance and the species abundance curve [50, 69]. It should be mentioned, however, that the species abundance curves do not contain a clear inflection point that could be used to define rare species objectively compared to the use of a predetermined threshold [e.g. 0.1% abundance]. Instead, the curve often appears to be a monotonic, log-normal decrease with no obvious inflection points but with a sharp change in slope (Fig. 2). Hence, the threshold to define rare taxa may appear somewhat arbitrary even with a species abundance curve available, although we do recommend the TAD80-based methodology outlined above as a more robust and well-defined approach that normalizes different sequencing efforts between samples (see also below). This definition can readily be applied to other ecosystems to consistently define rare vs. abundant genomospecies.

To further understand the key functional and/or ecological differences between taxa that respond to disturbances by changes in their abundance, we tested the disturbance-specialization hypothesis that generalists are favored while specialists are disfavored by disturbance. Our results provide support for this hypothesis based on the PS index, which is a direct way to measure taxon relative abundance changes across the entire dataset to define generalists vs. specialists. Our conclusion that generalists are more favored by disturbance events than specialists is also consistent with recent studies based on laboratory mesocosms [20]. Specifically, Chen and colleagues concluded that generalist taxa are more metabolically flexible. Consistently, we found that generalists have, on average, smaller and more compact genomes, which could provide an efficient metabolic strategy and a selective advantage during changing conditions, or alternately result in a lack of ability to respond to disturbance either positively or negatively. The smaller genome size is also consistent with the Black Queen Hypothesis that has been used to explain the ecological success of streamlined genomes through outsourcing of functions [70]. Further, we found that generalists have a slightly higher fraction of their genome devoted to extracellular protein functions compared to specialists, which could be another reason why generalists were favored by disturbance. That is, extracellular proteins represent mostly enzymes that degrade or transport public goods that presumably remain abundant throughout the disturbance. However, it should be also mentioned that in the winter disturbances and in the spring phytoplankton turnover, the abundant, resistant [i.e. their abundances are not affected by the disturbance] genomospecies were found to be largely specialists, which is not consistent with the disturbance-specialization hypothesis, further highlighting that each disturbance event may select for distinct organisms.

There is no standard or universally accepted definition for generalist/specialist except for Levins’ niche breadth index [54, 55] and the PS Index [56]. We employed the PS index because it allows abundant and widespread species to be also classified as habitat specialists when they show relatively high abundance in a single category of samples (so prevalence in more samples matters in addition to their abundance or absolute abundance) [50, 57]. We obtained largely consistent results to those reported above with the PS index when we used the PS implementation in the MicroNiche package, as well as with Levins’ niche breadth definition (Fig. S18) [50]. Further, the PS index, based on absolute abundance data, is commonly used in macroecology compared to Levins’ definition [57]. We used relative abundances of microbial taxa as compared to the absolute abundance typically used in macroecology since absolute abundances were not available for our datasets. Performing this type of analysis with absolute abundances and a more detailed measurement of the nutrient availability in each sample in future studies could further corroborate the conclusions presented here.

Our findings and conclusions are sensitive to the definition of rare genomospecies represented by MAGs. For example, the sequencing effort affects the limit of detection and the species abundance curve, and thus our definition, which was derived from the curve. Sequencing effort could also affect the PS index (generalist vs. specialist taxa), as with more sequencing additional genomes could become detectable. Under-sampling (in terms of sequencing coverage) of microbial communities, especially in complex environmental settings, also leads to poor assembly and MAG recovery. Nonetheless, we do not believe that these limitations substantially affected our conclusions. Specifically, when we used all metagenome reads (without subsampling to obtain the same sequencing effort across datasets), we obtained a total of 412 dereplicated MAGs compared to 198 MAGs that were obtained with the subsampled datasets. The total relative abundance of all 412 MAGs ranged between 8.4% and 37.9%. That is, the additional 214 MAGs were mostly abundant in a few of the disturbance events (specifically, 1, 2, and 3), which also explained why these MAGs were not recoverable in the subsampled datasets. Therefore, the effect of the additional MAGs on the definition of rare taxa or on what fraction of the community was represented by MAGs was rather limited [e.g. definition threshold of ~0.05% instead of the 0.1% used in many subsampled datasets; Fig. S14]. With respect to the PS index, we expected that the sequence effort limitation would apply evenly to all samples and rare taxa, and thus should not significantly affect the PS index values for most taxa and our derived conclusions. It would be valuable to see if our interpretations change with higher sequencing coverage and absolute abundance measurements in future studies as well as extending this research to other, more complex environments such as soils. Finally, a few abundant taxa with high intra-specific diversity are not well represented among our MAGs, such as the SAR11, rendering assembly of representative MAGs to be challenging. Hence, our MAG collection is phylogenetically biased with respect to disturbance-responsive-taxa although we anticipate that this bias has minor effects—if any—on our derived conclusions.

In conclusion, we found microbial communities in the temperate coastal ocean in the Southeast USA to exhibit characteristics of both resistance and resilience against natural disturbances (depending on whether the corresponding populations were abundant and remained abundant or became rare during the event and returned to abundant after the event, respectively), and provide evidence in support of the disturbance-specialization and the insurance hypotheses. Further, we provided a new approach based on the species abundance curve to define rare vs abundant populations as well as generalists vs. specialists based on a time-series metagenomic sampling. These definitions and approaches will be helpful for future studies to clarify the role and the importance of the rare biosphere. Collectively, the findings presented here advance our understanding of the natural microbial community response to disturbance, and thus, could be useful for modeling and monitoring microbial diversity, including microbiome rescue [8] in a changing world.

## Supplementary Material

Supplementary_materials__wraf129

Extended_data_S1_dilution_rare2abunt

Extended_data_S2_DOM_rare2abunt

## Data Availability

Raw metagenomic reads are available as NCBI BioProjects PRJNA643505 and PRJNA934397 as well as NCBI Projects 441 405–441 416 and 16S rRNA gene libraries are available as PRJNA309156.
