# Supplementary material for: Quantifying the contribution of the rare biosphere to natural disturbances
Source: ISME J. 2025 Jun 26;19(1):wraf129. doi: 10.1093/ismejo/wraf129 (PMC12286922; doi:10.1093/ismejo/wraf129)
Supplement: Supplementary_materials__wraf129 [file supplementary_materials__wraf129.pdf]

Supplementary Material

Quantifying the contribution of the rare biosphere to the microbial community response during natural disturbances

Jianshu Zhao, Genevieve Brandt, Jessica L. Gronniger, Zhao Wang, Jiaqian Li, Dana E. Hunt, Luis M. Rodriguez-R, Janet K. Hatt, Konstantinos T. Konstantinidis

Supplementary Materials and Methods

Mesocosm experiment design

The mesocosm experiment was designed to examine controlled disturbances without the background of environmental variability. We tested two key hurricane-associated processes with significant potential for altering the microbial community: (1) a dilution of the microbial community from the influx of freshwater and (2) a phytoplankton-bloom, which would increase labile dissolved organic matter. These two processes were not only the most marked changes induced by hurricane Florence (2018) at our study site, but are also commonly observed[1-3]. The environmental data from hurricane Florence informed the design of 5-day experimental manipulations to test the effects of dilution of the bacterioplankton community that mimics hurricane washout effects (without salinity changes), and a daily addition of diatom lysate to mimic labile organic matter from a hurricane-induced bloom. The experiment was conducted in an environmental chamber that aimed to simulate *in situ* environmental conditions. Experimental manipulations involved triplicate carboys assigned to either control, a 1:10 dilution of the bacterioplankton community using 0.2 μm filtered PICO seawater, and a daily DOM addition treatment of 10 μM of lysate from a culture of the bloom-forming diatom *Skeletonema costatum*, representing a projected ~45% increase in daily primary production relative to non-hurricane conditions. Experimental conditions included controls, an initial 90% dilution in 0.22 µm filtered coastal seawater and a daily addition of diatom-derived DOM. The DOM was obtained from a lab-grown culture of *Skeletonema costatum* harvested during exponential growth phase on a 0.8 µm polycarbonate filter.

Harvested cells were suspended in autoclaved Nanopure water and lysed using 0.5 mm beads with the following protocol: samples were beaten at 4800 RPM for 30 seconds followed by a 2-minute incubation on ice, repeated for a total of 2 minutes of bead beating. This lysate was then 0.2 µm filtered and dissolved organic carbon content was quantified using a total organic carbon analyzer (TOC-L Shimadzu). DOM lysate was then aliquoted and diluted to working concentrations using autoclaved Nanopure water and frozen at -20 °C until use. Each treatment consisted of three replicated 20 L acid-washed, polycarbonate carboys. Carboys were filled at PICO (34.7181 °N 76.6707 °W) on July 28th, 2022. Water from 1 m below the surface filtered through a 200 *µ*m nylon mesh screen to avoid the presence of large zooplankton was pumped into the carboys. The water for 90% dilution water was filtered from the same location the day prior using 0.22 *µ*m polythersulfone filters (Steripak). Carboys were incubated in environmental chambers set to ambient seawater temperature and were constantly purged with air filtered through a charcoal filter (Whatman Carbon-Cap 150) at ~10 L/min and stirred at ~200 rpm. Light/dark cycle was set at 12:12 hours at a light level of ~100 µE m^{-2} s^{-1}. Carboys were incubated in the environmental chamber for five days, during which time the DOM treatments received a daily addition of *Skeletonema costatum* lysate (net 10 *µ*M carbon addition) in order to replicate semi-continuous input of phytoplankton bloom-associated DOM.

DNA extraction

Samples for nucleic acid extraction were collected by filtering ~1 liter of seawater through a 0.22-micron Sterivex filter (Millipore, Darmstadt, Germany) and the filters were stored

at -80 °C until DNA extraction. Genomic DNA was extracted using the phenol-chloroform lysis supplemented with bead beating (60 seconds) and then subsequently cleaned using the Zymo OneStep PCR inhibitor removal kit. Extracted DNA was quantified using a Nanodrop ND-100 before sequencing as described in the main text.

16S rRNA amplicon sequencing and analysis

16S rRNA gene amplicons from each sample were sequenced using the primers targeting the V3-V4 region of the bacterial and archaeal 16S rRNA genes as described previously[4]: 16S F V3, CCTACGGGNGGCWSCAG; and 16S R V4, GGACTACNVGGGTWTCTAAT. USEARCH v11.0.667 was used for quality control and merging of paired-end reads. We first trimmed low-quality bases from the sequences using a 10-basepair window with a Q30 running-quality threshold. Paired-end sequences with a >=10 basepair overlap and no mismatches were then merged. We performed a final filtering step to discard low-quality merged sequences with a length of <400 bp and/or a maximum expected error of >1. Operation Taxonomy Units (OTUs) were identified using the UPARSE algorithm in USEARCH[5]. Singletons were removed before generating the OTU table. Taxonomic classification of OTU sequences was performed using SINTAX (-sintax_cutoff 0.8, a significance threshold similar to the 50% bootstrap cutoff accuracy of the RDP naïve classifier) against the SILVA v138.1 database in USEARCH[6, 7]. MacQIIME v1.9.1 was used for rarefaction, alpha diversity, beta diversity and community composition analysis[8].

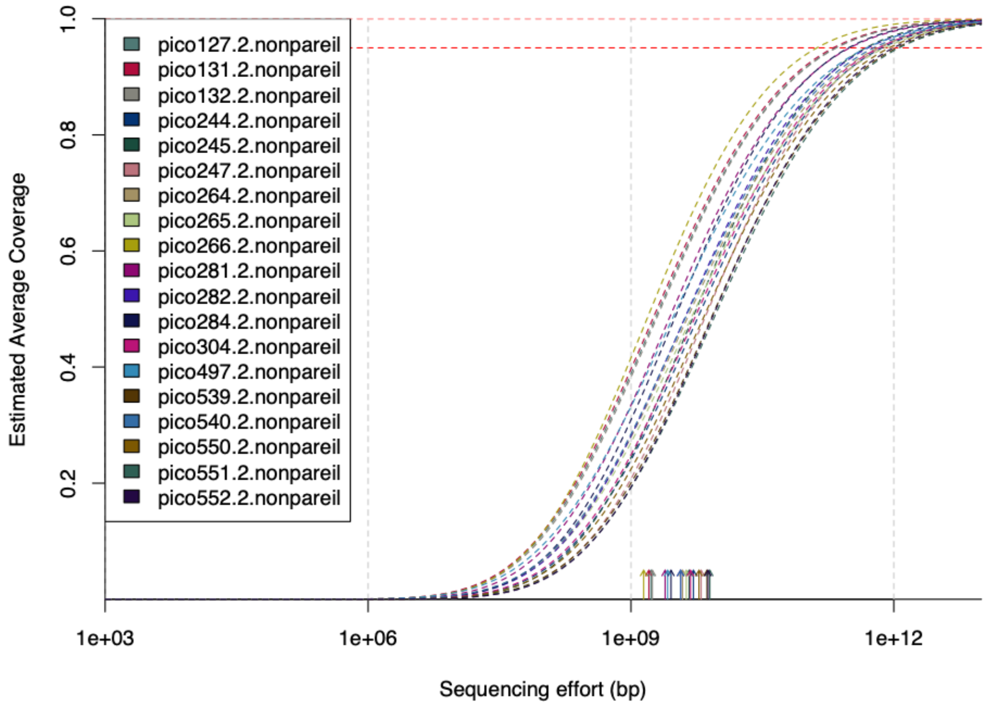

Figure S1. Nonpareil curves showing the coverage of subsampled time series metagenomes. Dashed lines show the estimated average coverage (y-axis) as sequencing efforts increases (x-axis) with current coverage labeled with an arrow for each sample; Red dashed lines represent the projections for 95% and 99% coverage (horizontal dashed lines on the top). Only reverse reads were used for coverage estimation as suggested previously [9] for sequences used to not be linked/associated to each other (independent observations); forward reads showed similar curves for each sample (not shown).

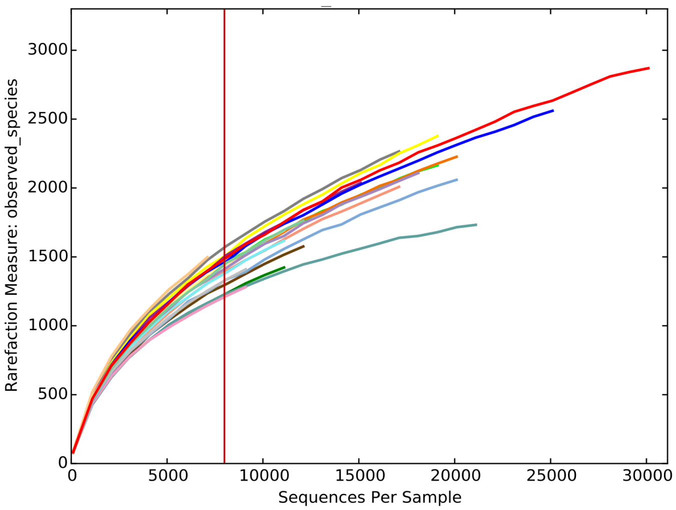

Figure S2. Rarefaction curves for extracted 16S rRNA gene-carrying reads from time series metagenomes. Each curve represents a sample. The vertical red line shows the number of reads subsampled (8000) for downstream analysis.

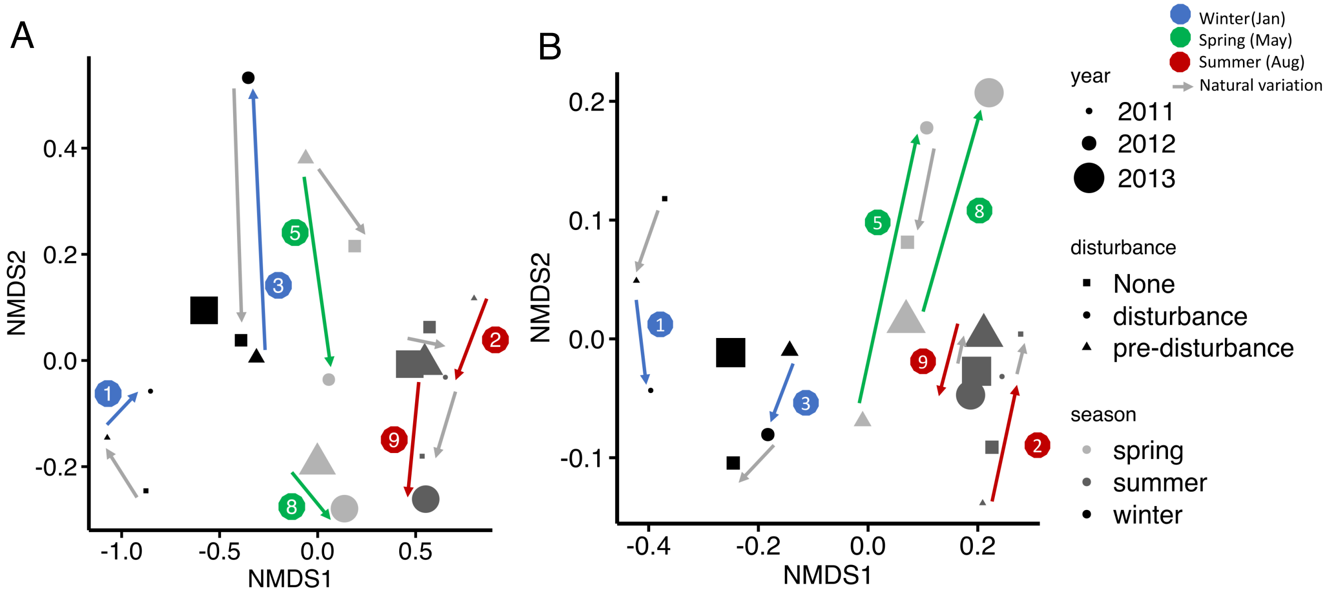

Figure S3. NMDS plot of 16S rRNA gene amplicon sequences (a) and 16S-carrying reads from time series metagenomes (b). Arrows show the direction that the microbial community composition changed by each disturbance event. Grey arrows show the natural variation of metagenomic composition.

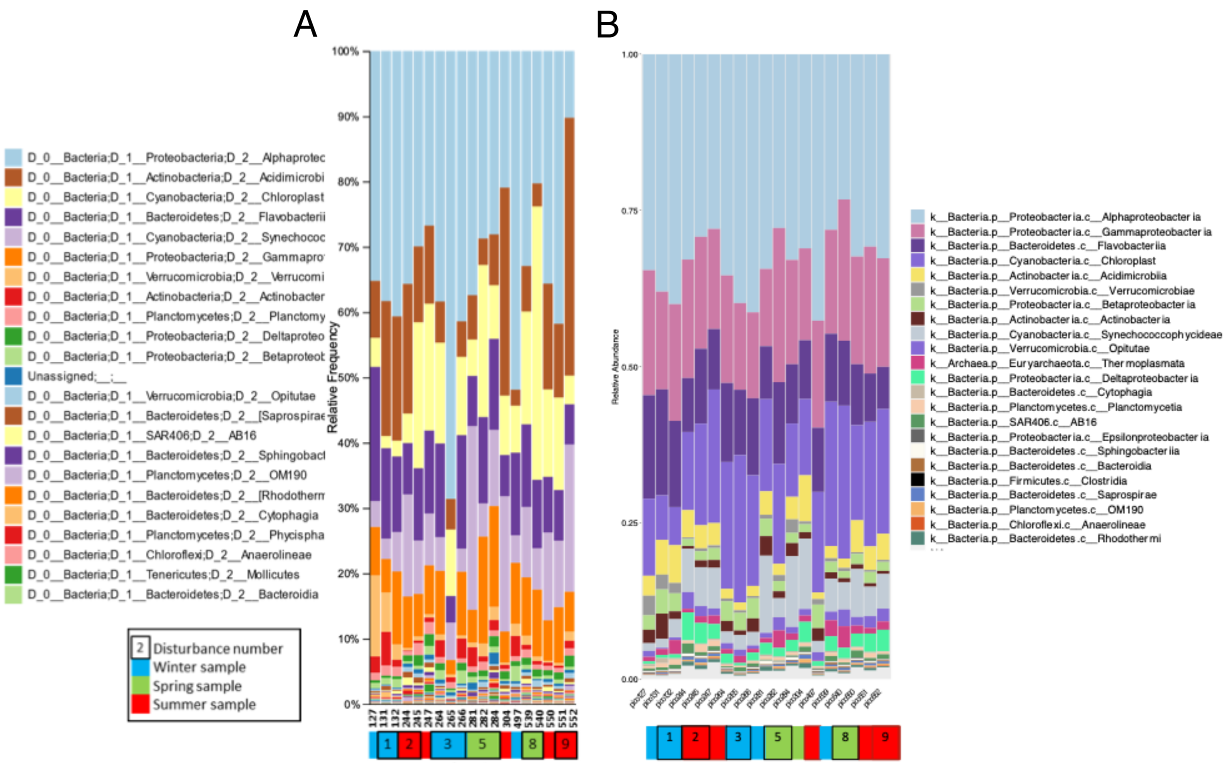

Figure S4. Class level microbial community composition (relative abundance) for 16S rRNA gene amplicon sequences (a) and 16S-carrying reads from time series metagenomes (b). Each column represents a sample, with sample details provided by the color box below the column. Disturbance events are labelled by the same number as in Figure 1a. See Figure 1a for detailed explanation for each disturbance event.

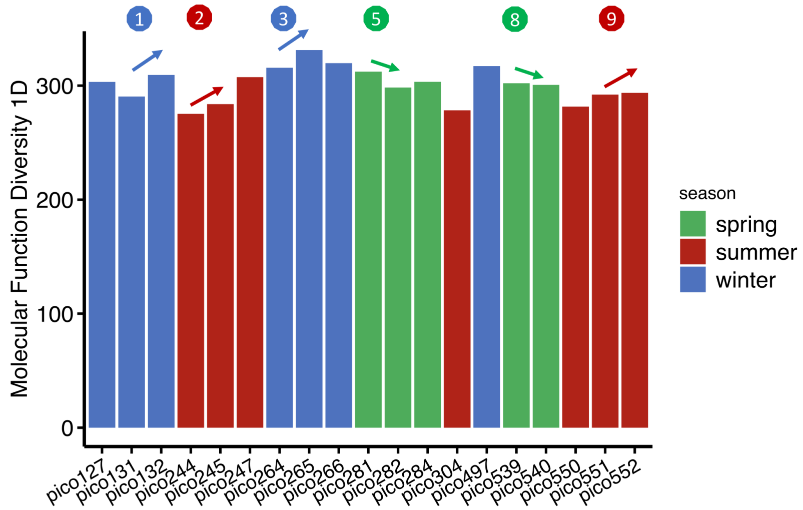

Figure S5. Changes in molecular functional diversity of time series metagenomes as revealed by mapping reads to annotated functional pathways (See Materials and Methods for details). Disturbances are numbered and shown with arrows.

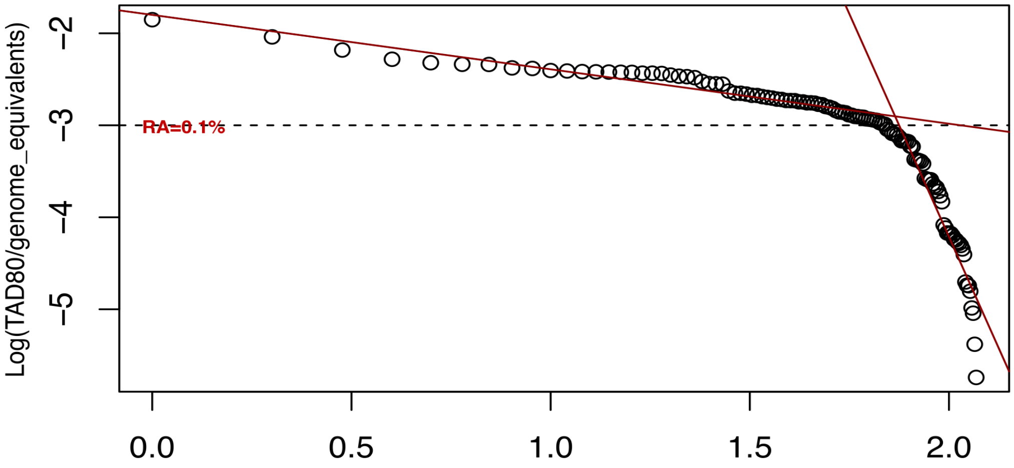

Figure S6. Log-log fitting of normalized sequence coverage depth vs. abundance rank for all time-series MAGs. Two linear fittings were performed using MAGs with normalized coverage depth greater and less than 0.1%, respectively ($R^2 > 0.7$). A clear difference in the slope of the two fitted lines indicate a sharp decrease for normalized coverage depth around 0.1%.

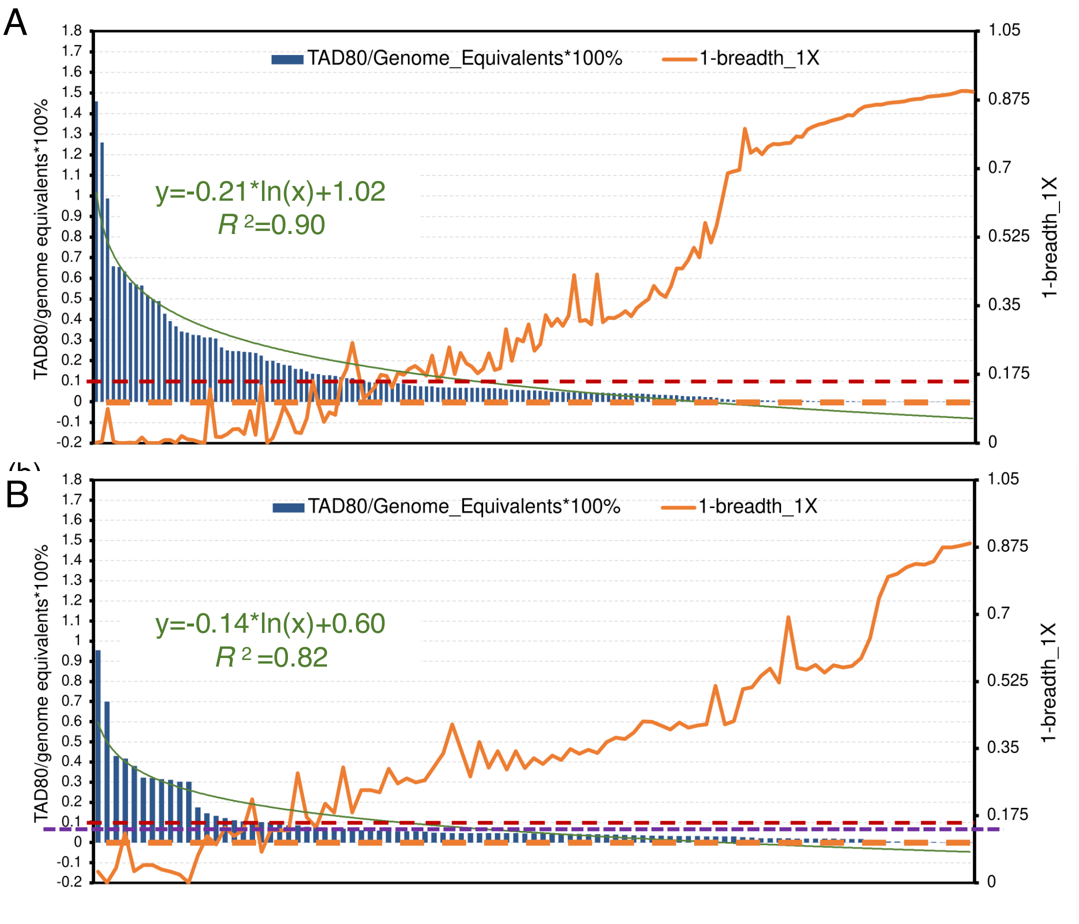

Figure S7. MAG coverage depth (left y axis, blue bar) and coverage breadth (right y axis, orange line, shown as 1- coverage breadth) distribution for two metagenomic samples, pico284 (a) and pico247 (b). This figure shows two additional examples and consistent patterns to those observed in Figure 2. The X-axis is the MAG abundance rank based on coverage depth (TAD80 normalized by genome equivalents). Dashed red and orange lines represent a normalized coverage depth of 0.1% and coverage breadth of 0.1, respectively. The green line is a log fitting of coverage depth vs. rank with the corresponding function shown above it. Before subsampling, pico284 had similar sequencing depth as pico127 (Figure 2), while pico247 is the shallowest sequenced sample of the three, thus there are a smaller number of MAGs showing high coverage depth (i.e., less reads could be mapped to the dereplicated 198 MAGs). We use an RA threshold of 0.08% in this case since there is a sharp decrease in coverage breadth round 0.08% (purple instead of red dashed lines).

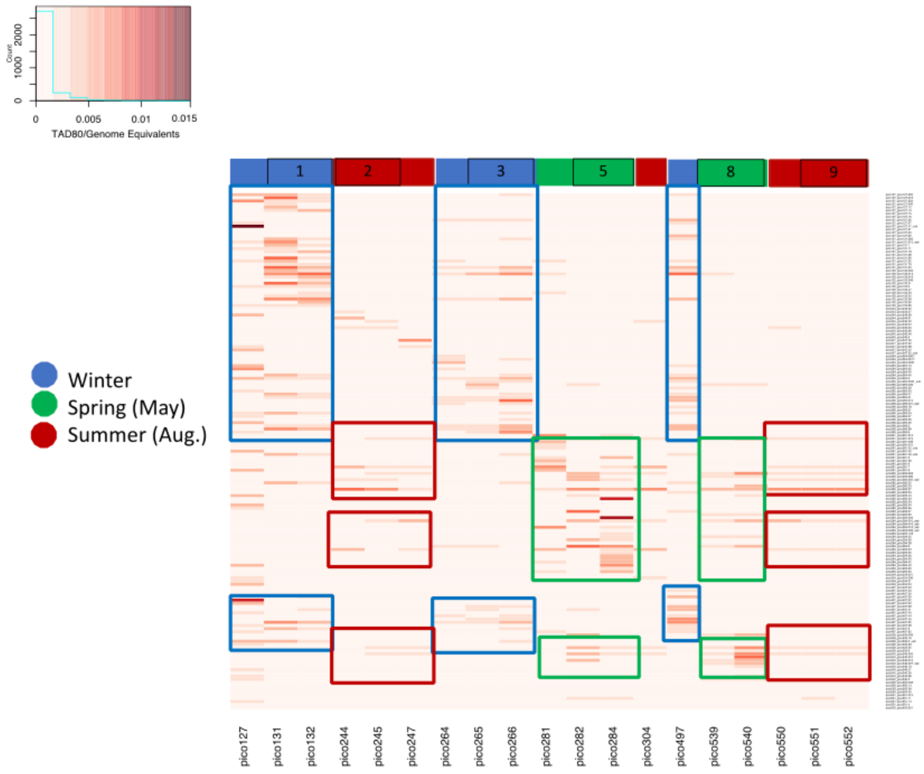

Figure S8. Heatmap of time series MAG relative abundance. Each row represents a MAG while each column represents a sample (see key for sample designation by color). Disturbance events are labelled by a number in the top of the heatmap as in Figure 1a. See Figure 1a for a detailed explanation for each disturbance event. Labeled boxes represent MAGs that showed clear responses in terms of their relative abundance changes for each event.

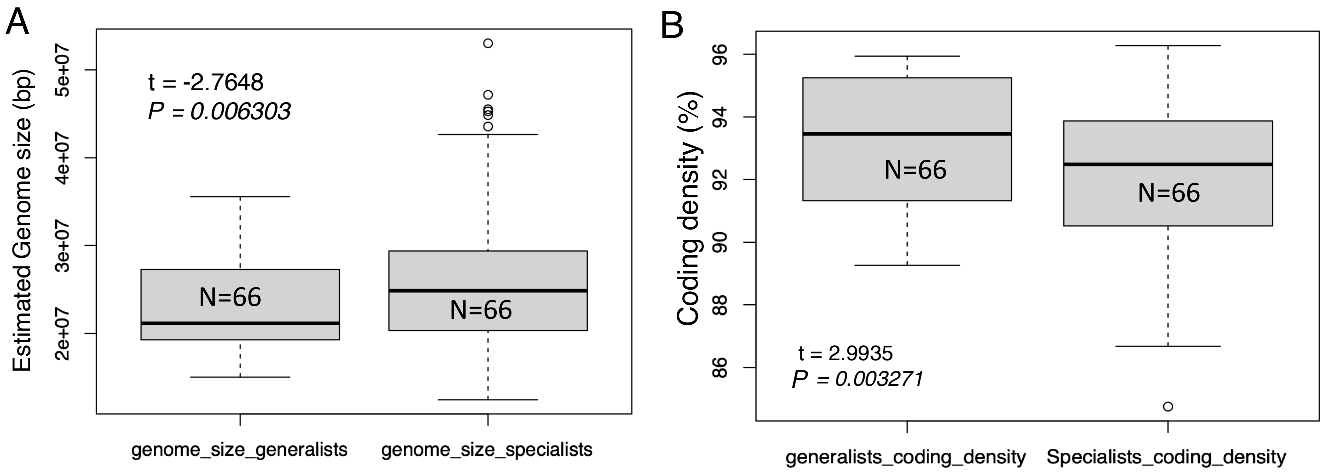

Figure S9. Estimated genome size (a) and coding density (b) differences between generalist vs. specialist MAGs. Both the T-test and Mann–Whitney test were significant (note the *P* values shown on the graphs).

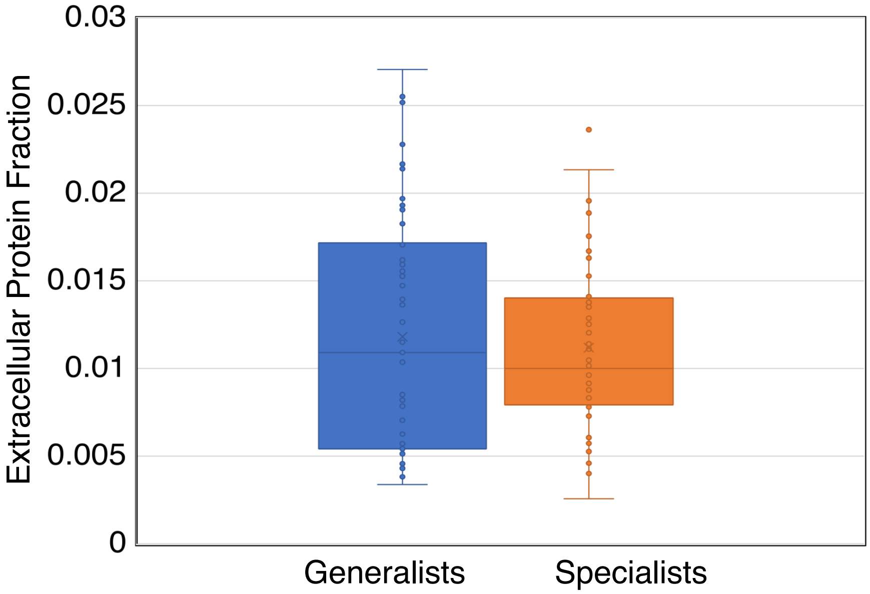

Figure S10. Proteins predicted to be extracellular, expressed as a fraction of the total genes in the in the genome (y-axis), for generalist (Blue) and specialist (Orange) MAGs. The difference is significant at $P < 0.05$ (Mann-Whitney test).

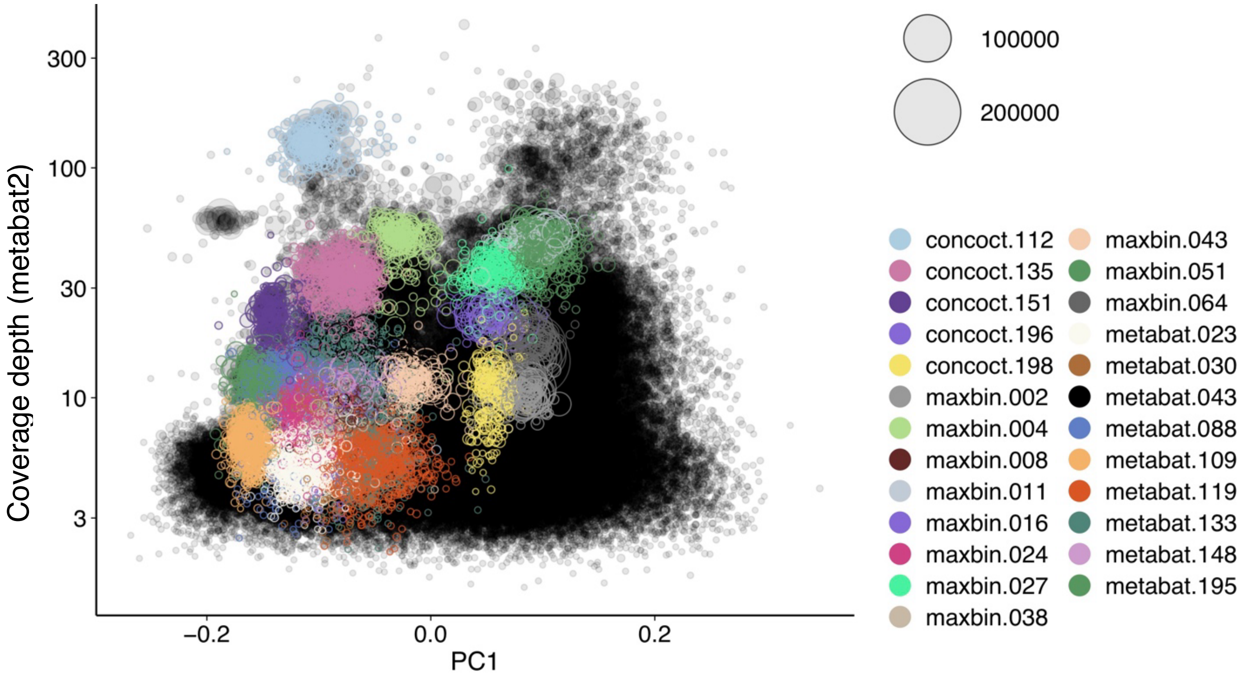

Figure S11. Contig coverage depth vs. principal component 1 of tetranucleotide frequencies for sample pico127 showed that each recovered MAG represents a largely distinct sequence cluster of contigs. Binned contigs by 3 different pieces of binning software (MaxBin2, MetaBAT2 and CONCOCT) are labelled by software name and MAG ID. Contigs that were binned into the same MAG are labeled by the same color. Each circle represents a contig in the assembly while the size of each circle represents the contig length.

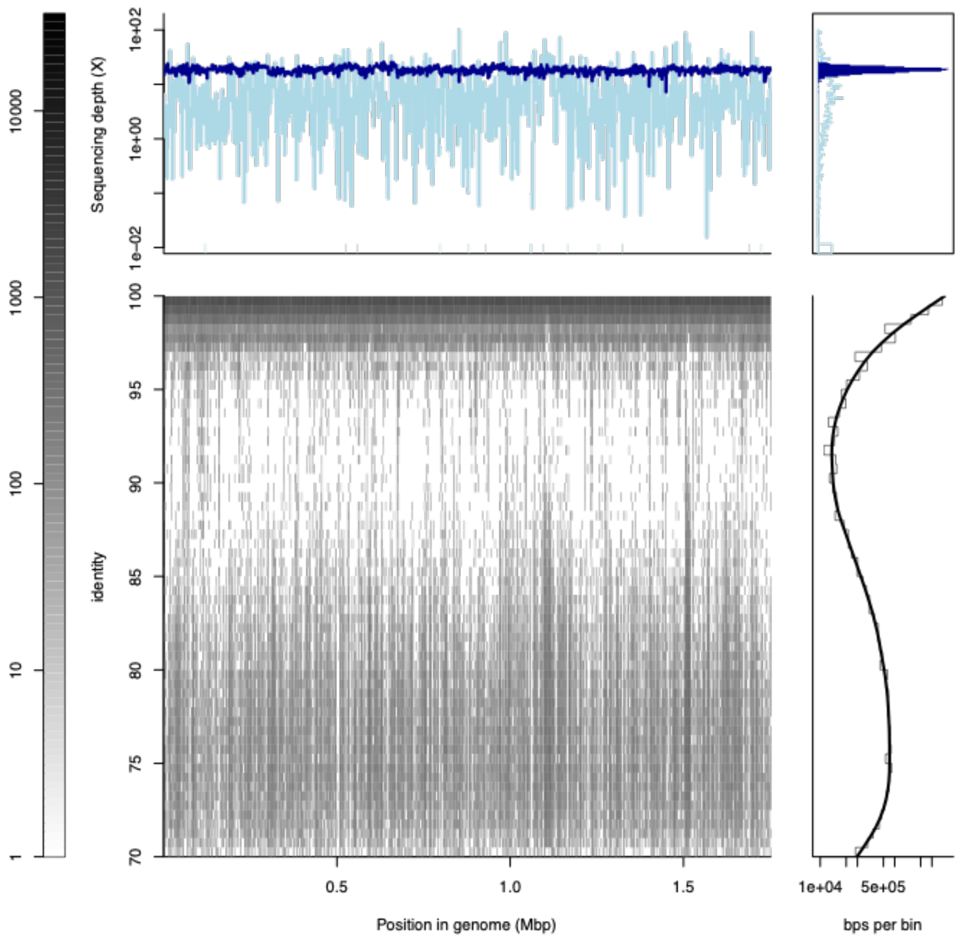

Figure S12. Recruitment plot of one MAG from sample pico497 as an example that the recovered MAGs represent sequence discrete populations. The reference MAG represents a sequence-discrete population in the PICO 497 metagenome because there are many reads mapping on the MAG with >95% nucleotide, contrasting with reads showing 85-95% identity that are relatively sparse. Average sequence coverage depth of this MAG is ~23X. An interactive version of this plot is available through: https://github.com/jianshu93/RecruitmentPlot_blast/blob/main/example_out/pico497.23.html.zip.

All Recruitment plots for all MAGs of each sample are available here:

https://github.com/jianshu93/RecruitmentPlot_blast/tree/main/example_out/pico_rec_plot_2 and here:

https://github.com/jianshu93/RecruitmentPlot_blast/tree/main/example_out/pico_rec_plot.

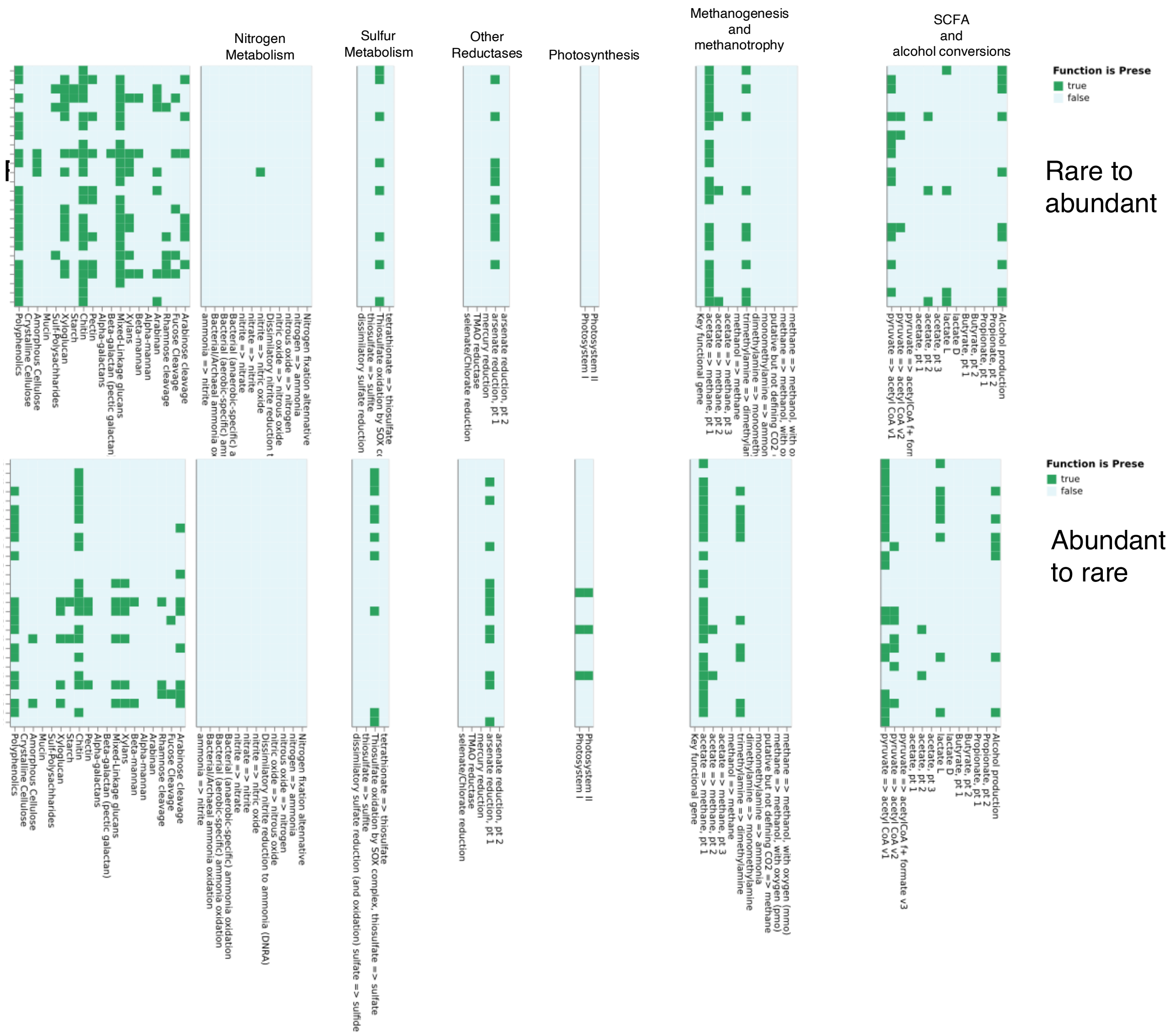

Figure S13. Metabolic pathways encoded in the genome of abundant-to-rare and rare-to-abundant MAGs for disturbance event 5 based on DRAM.

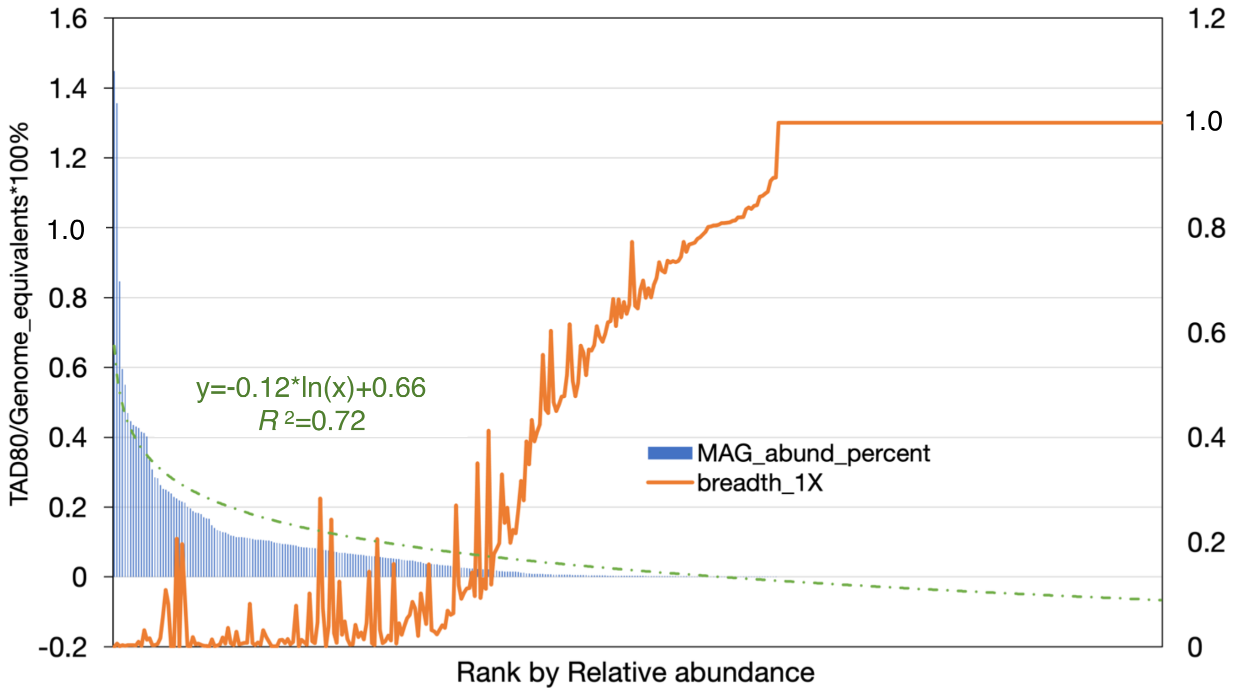

Figure 14. MAG sequence depth (left y-axis, blue bar) and breadth (right y-axis, orange line, shown as 1- coverage breadth) coverage distribution for PICO127 before subsampling shows a similar fitted line as the subsampled dataset shown in Figure 2 and a slightly shifted abundance threshold for defining rare taxa (around 0.05).

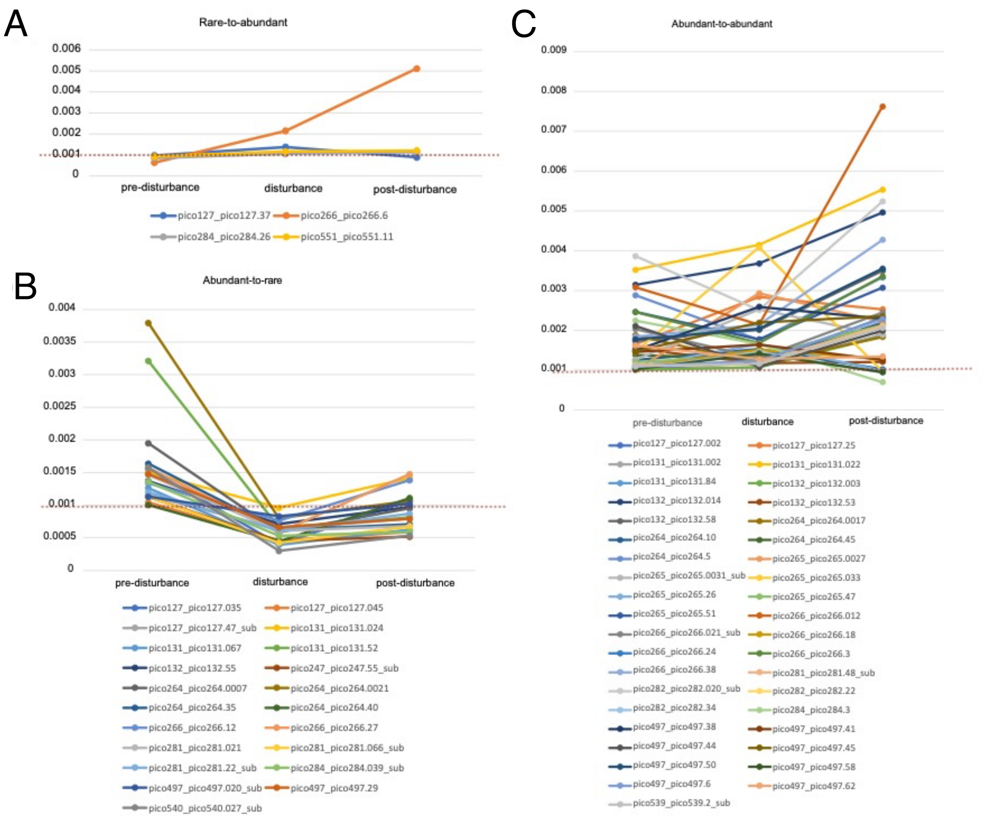

Figure S15. Relative abundance of MAGs assigned to rare-to-abundant (a), abundant-to-rare (b) and abundant-to-abundant (c) categories for disturbance 3 (in Figure 1a). Relative abundance (y-axis) was estimated as TAD80 sequence depth divided by genome equivalents to normalize for any average genome size differences between the samples as described in the Materials and Methods section. The red dashed line represents the threshold used to define rare taxa. Note the higher similarity in abundances between the pre- and post-disturbance samples relative to the disturbance sample, which indicates that stochastic processes have limited effect in identifying MAGs that change abundance categories due to the disturbance event.

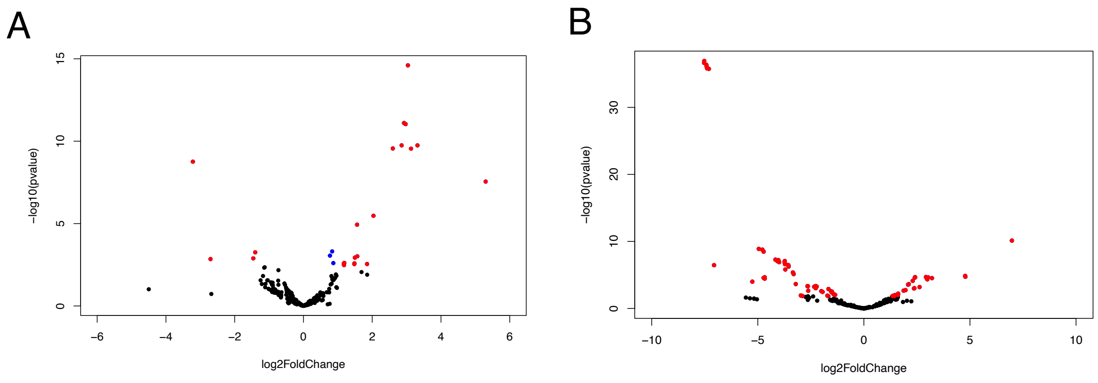

Figure S16. (a) DESeq2 volcano plot [10] for MAGs with significant abundance changes for the DOM addition mesocosm treatment. (b) Same plot with (a) but for the dilution treatment. Points are labelled in blue if $P_{adj}<0.05$, red if log2 Fold_Change>1 and $P_{adj}<0.05$. The abundance of each MAG was defined based on its sequencing depth by mapped reads and represented the input values to DESeq2. The DeSeq comparison was done between abundances in the control (n=3 replicates) and treatment (n=3 replicates) metagenomes.

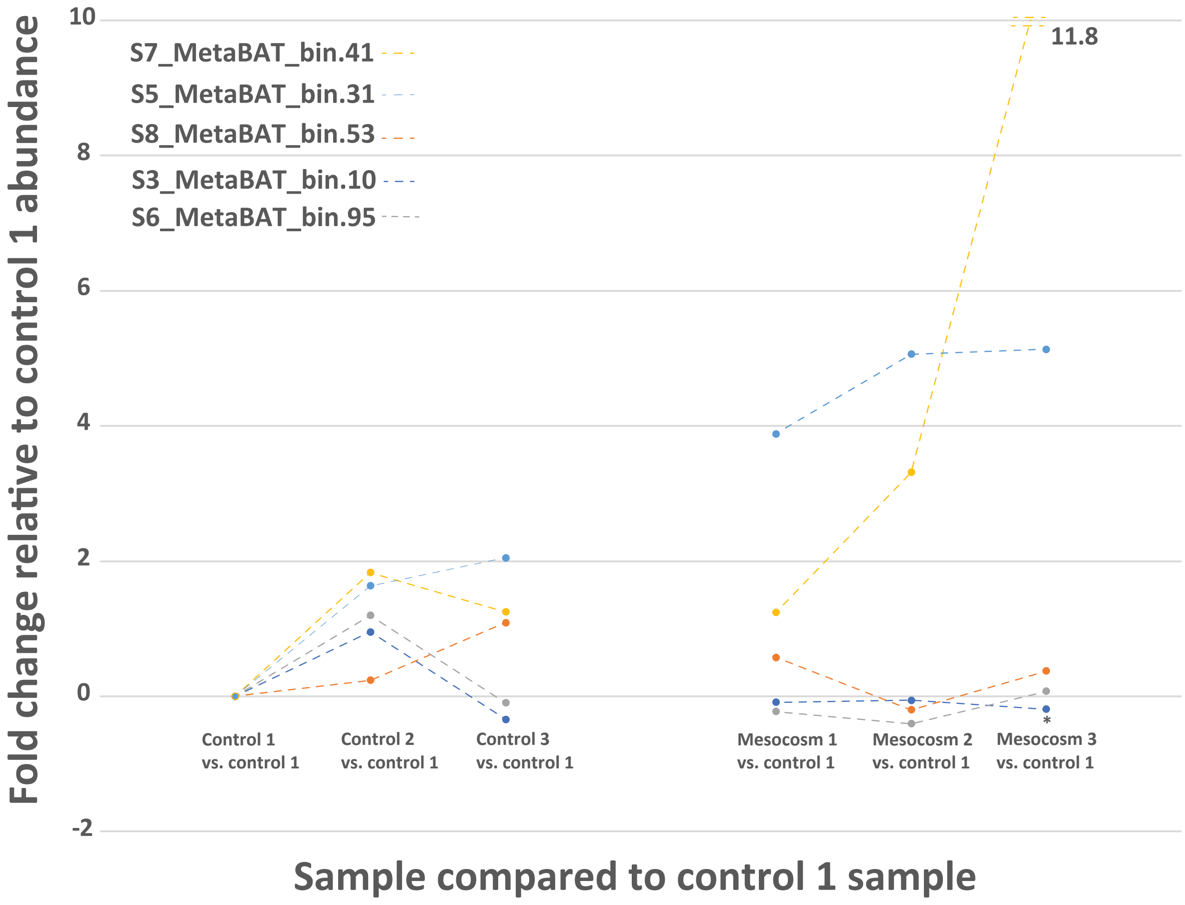

Figure S17. Abundance patterns of five field MAGs found to change in abundance (from rare to abundant) during disturbance event #5 (spring diatom bloom) in the DOC mesocosms. The abundance of each MAG (same species as the field MAG, >98% ANI) in the two control (#C2 and #C3) and the three DOM addition treatments (#M1, #M2 and #M3) was compared to its abundance in control #C1 to simulate the pairwise sample analysis performed for field MAGs based on before and after disturbance samples that is reported in the main text. The change in abundance relative to control #C1 is plotted (y-axis) against the sample compared to control #C1 (x-axis). The datapoints for each MAG have been connected with a dashed line to be easier to identify them. Note that two of the total five MAG showed increased abundance in the mesocosm treatment relative to the control (became abundant from rare) while the remaining three did not change in abundance category (remained rare).

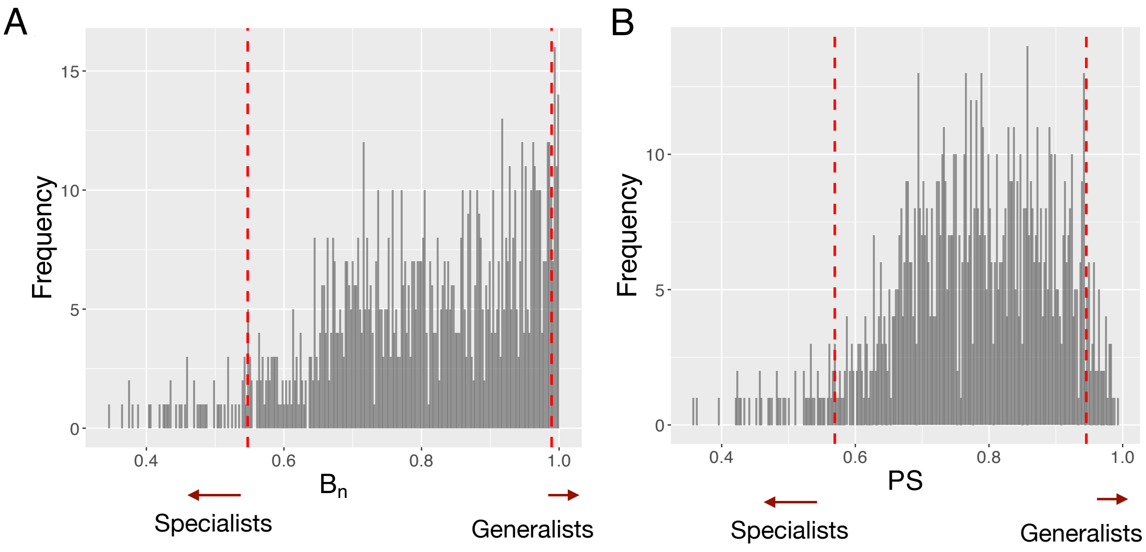

Figure S18. Levin's Breadth index (a) and PS index (b) in MicroNiche package to identify generalists and specialists. Each line represents a MAG and shows its frequency values (y-axis) in the categories of samples evaluated plotted against the estimate index value based on the frequency (x-axis). Note the high similarity in the distributions obtained (see main text for additional discussion).

Supplementary Tables

Table S1. Measured environmental variables for the time-series field samples. Bold lines represent samples during disturbance events. See Figure 1a for sample details of each event.

disturbance	Sample name	Temp	MLLW[a]	Salinity	Oxygen	OxygenSaturation	pH	DIC	Chloro	NH4	PO4	SiO4
	pico127	7.4	0.62	32	9.74	99.8	7.92	1971	3.61	390.63	0.05	1.79
**1**	**pico131**	**8.9**	**0.03**	**32**	**9.89**	**104.9**	**7.92**	**1967.62**	**4.1**	**167.46**	**0.04**	**1.92**
	pico132	9.9	0.89	35	9.37	103.5	7.93	2073.19	2.45	174.05	0.04	0.92
	pico244	29	1.13	34	6.55	103	8.08	2106.39	3.26	193.37	0.07	2.42
2	**pico245**	**27.8**	**0.31**	**35**	**5.85**	**90**	**8.02**	**2156.02**	**5.81**	**319.94**	**0.13**	**4.36**
	pico247	28	0.25	31	6.39	97.1	7.94	1986.3	7.96	218.94	0.03	13.95
	pico264	10	0.08	31	8.8	94.3	7.92	2096.6	3.11	73.76	0	2.83
3	**pico265**	**11.4**	**0.8**	**33.5**	**8.73**	**98.1**	**7.92**	**2145.7**	**2.4**	**303.85**	**0**	**2.05**
	pico266	9.6	0.2	33	9.16	99.2	7.89	2157.37	2.38	229.76	0.01	3.58
	pico281	21.1	-0.05	30.5	7.74	103.1	7.87	2060.23	5.65	153	0.06	8.25
5	**pico282**	**21.8**	**1.12**	**34**	**7.84**	**106.8**	**7.95**	**2157.2**	**3.86**	**130**	**0**	**3.64**
	pico284	22.3	0.94	33	7.39	102.8	7.92	2119.8	5.9	123.5	0.02	7.52
	pico304	29.1	0.36	33	6.74	107.2	8.01	2135.5	6.1	102	0	5.52
	pico497	10.1	0	32.75	7.99	86.9	7.9	2115.77	1.82	24.5	0.02	1.94
8	pico539	23	0.83	34.78	7.03	97.7	7.94	2111.17	3.02	22	0.05	1.55
	**pico540**	**24.7**	**0.18**	**34.57**	**6.65**	**95.1**	**7.95**	**2107.43**	**6.8**	**9**	**0.02**	**1.98**
	pico550	26.9	0.84	34.99	6.25	94.7	7.95	2099.73	5.52	29.5	0.03	4.52
9	pico551	28.3	0.48	35.41	5.73	89	7.97	2115.97	6.71	95	0.05	6.76
	**pico552**	**26.9**	**0.89**	**34.97**	**6.26**	**94.7**	**7.95**	**2106.8**	**5.46**	**53**	**0.03**	**5.29**

[a]mean of the two low tides.

Table S2. A summary of the key environmental parameters characterizing each of the six disturbance events studied.

**Event number**	**Key changes**
1	58% less ammonium
2	65% more ammonium, 80.2% more silicates and 78.2% more chlorophyll A
3	3.1 times more ammonium
5	116% more NOx, 55.9% less silicates and 31.7% less chlorophyll A
8	observed 100.1% more chlorophyll A and 59% less ammonium
9	220.3% more ammonium, 49.6% more silicate and 20.2% more chlorophyll *a*, 1.5 ^{0}C higher

Table S3. Quality and classification of the high quality, dereplicated time series MAGs. Quality was based on CheckM while classification was based on GTDB-tk v1.1 against GTDB v207.

Genome name	Completeness	Contamination	Classification
pico127_pico127.002	92.02	5.44	d__Bacteria;p__Proteobacteria;c__Alphaproteobacteria;o__Rhodobacterales;f__Rhodobacteraceae;g__Planktomarina;s__Planktomarina temperate
pico127_pico127.018	75.91	7.94	d__Bacteria;p__Verrucomicrobiota;c__Verrucomicrobiae;o__Verrucomicrobiales;f__Akkermansiaceae;g__UBA985;s__
pico127_pico127.023	92.47	6.38	d__Bacteria;p__Proteobacteria;c__Gammaproteobacteria;o__Pseudomonadales;f__Porticoccaceae;g__HTCC2207;s__HTCC2207 sp002685195
pico127_pico127.035	90.35	8.53	d__Bacteria;p__Actinobacteriota;c__Actinomycetia;o__Actinomycetales;f__Microbacteriaceae;g__Pontimonas;s__
pico127_pico127.045	93.35	4.69	d__Bacteria;p__Proteobacteria;c__Gammaproteobacteria;o__Pseudomonadales;f__HTCC2089;g__UBA4582;s__UBA4582 sp002389265
pico127_pico127.062	82.36	5.96	d__Bacteria;p__Actinobacteriota;c__Actinomycetia;o__Nanopelagicales;f__S36-B12;g__UBA6154;s__
pico127_pico127.12	80.09	4.08	d__Bacteria;p__Bacteroidota;c__Bacteroidia;o__Flavobacteriales;f__Flavobacteriaceae;g__Algibacter_B;s__
pico127_pico127.14	89.57	4.8	d__Bacteria;p__Actinobacteriota;c__Acidimicrobiia;o__Acidimicrobiales;f__Ilumatobacteraceae;g__Ilumatobacter_A;s__Ilumatobacter_A sp002711735
pico127_pico127.15	89.93	3.47	d__Bacteria;p__Bacteroidota;c__Bacteroidia;o__Flavobacteriales;f__Flavobacteriaceae;g__Winogradskyella;s__
pico127_pico127.16	88.44	0.82	d__Bacteria;p__Bacteroidota;c__Bacteroidia;o__Flavobacteriales;f__Flavobacteriaceae;g__MAG-120531;s__
pico127_pico127.19	77.92	0	d__Archaea;p__Thermoplasmatota;c__Poseidoniia;o__Poseidoniales;f__Poseidoniaceae;g__MGIIa-L1;s__MGIIa-L1 sp002506275
pico127_pico127.25	90.15	1.26	d__Bacteria;p__Proteobacteria;c__Alphaproteobacteria;o__Rhodobacterales;f__Rhodobacteraceae;g__LFER01;s__LFER01 sp001642945
pico127_pico127.37	78.24	1.7	d__Bacteria;p__Proteobacteria;c__Alphaproteobacteria;o__Rhodobacterales;f__Rhodobacteraceae;g__Amylibacter;s__Amylibacter sp900197625
pico127_pico127.47_sub	77.6	1.88	d__Bacteria;p__Bacteroidota;c__Bacteroidia;o__Flavobacteriales;f__Schleiferiaceae;g__UBA10364;s__UBA10364 sp003045825
pico127_pico127.51	86.6	0.93	d__Bacteria;p__Proteobacteria;c__Gammaproteobacteria;o__Pseudomonadales;f__Pseudohongiellaceae;g__UBA9145;s__UBA9145 sp003483155
pico127_pico127.64	86.83	2.96	d__Bacteria;p__Bacteroidota;c__Bacteroidia;o__Flavobacteriales;f__Schleiferiaceae;g__UBA10364;s__UBA10364 sp002387615
pico127_pico127.69	81.75	1.24	d__Bacteria;p__Bacteroidota;c__Bacteroidia;o__Flavobacteriales;f__Flavobacteriaceae;g__CAU-1491;s__
pico131_pico131.002	96.06	6.69	d__Bacteria;p__Proteobacteria;c__Alphaproteobacteria;o__Rhodobacterales;f__Rhodobacteraceae;g__Planktomarina;s__Planktomarina temperate
pico131_pico131.013_sub	92.13	9.12	d__Bacteria;p__Actinobacteriota;c__Actinomycetia;o__Nanopelagicales;f__S36-B12;g__UBA6154;s__
pico131_pico131.022	88.06	6.49	d__Bacteria;p__Proteobacteria;c__Alphaproteobacteria;o__Puniceispirillales;f__Puniceispirillaceae;g__Puniceispirillum;s__
pico131_pico131.024	91.4	6.18	d__Bacteria;p__Bacteroidota;c__Bacteroidia;o__Flavobacteriales;f__Schleiferiaceae;g__UBA10364;s__UBA10364 sp003045825
pico131_pico131.047	86.21	3.71	d__Bacteria;p__Proteobacteria;c__Gammaproteobacteria;o__Pseudomonadales;f__Porticoccaceae;g__HTCC2207;s__
pico131_pico131.048	82.69	4.69	d__Bacteria;p__Actinobacteriota;c__Actinomycetia;o__Actinomycetales;f__Microbacteriaceae;g__Pontimonas;s__
pico131_pico131.067	75.61	10.7	d__Bacteria;p__Proteobacteria;c__Gammaproteobacteria;o__Pseudomonadales;f__Pseudohongiellaceae;g__OM182;s__OM182 sp003482475
pico131_pico131.1	98.11	4	d__Bacteria;p__Bacteroidota;c__Bacteroidia;o__Flavobacteriales;f__Crocinitomicaceae;g__Crocinitomix;s__

pico131_pico131.11	97.43	0.74	d__Bacteria;p__Bacteroidota;c__Bacteroidia;o__Flavobacteriales;f__Flavobacteriaceae;g__MS024-2A;s__
pico131_pico131.18	88.79	5.73	d__Bacteria;p__Actinobacteriota;c__Acidimicrobiia;o__Acidimicrobiales;f__Ilumatobacteraceae;g__Ilumatobacter_A;s__Ilumatobacter_A sp002711735
pico131_pico131.28	92.57	0.83	d__Bacteria;p__Bacteroidota;c__Bacteroidia;o__Flavobacteriales;f__Flavobacteriaceae;g__MAG-120531;s__
pico131_pico131.30	87.02	0.33	d__Bacteria;p__Bacteroidota;c__Bacteroidia;o__Flavobacteriales;f__Flavobacteriaceae;g__;s__
pico131_pico131.50	87.93	9.84	d__Bacteria;p__Proteobacteria;c__Gammaproteobacteria;o__SAR86;f__SAR86;g__GCA-2707915;s__
pico131_pico131.52	77.93	5.87	d__Bacteria;p__Proteobacteria;c__Gammaproteobacteria;o__Burkholderiales;f__Methylophilaceae;g__BACL14;s__
pico131_pico131.78	81.42	1.57	d__Bacteria;p__Bacteroidota;c__Bacteroidia;o__Flavobacteriales;f__Crocinitomicaceae;g__UBA4466;s__
pico131_pico131.84	85.91	2.32	d__Bacteria;p__Proteobacteria;c__Gammaproteobacteria;o__Burkholderiales;f__Methylophilaceae;g__BACL14;s__
pico132_pico132.003	91.08	5.93	d__Bacteria;p__Proteobacteria;c__Alphaproteobacteria;o__Rhodobacterales;f__Rhodobacteraceae;g__Planktomarina;s__Planktomarina temperate
pico132_pico132.014	86.1	8.79	d__Bacteria;p__Proteobacteria;c__Alphaproteobacteria;o__Puniceispirillales;f__Puniceispirillaceae;g__Puniceispirillum;s__
pico132_pico132.019	91.97	7.36	d__Bacteria;p__Actinobacteriota;c__Acidimicrobiia;o__Acidimicrobiales;f__Ilumatobacteraceae;g__Ilumatobacter_A;s__Ilumatobacter_A sp002711735
pico132_pico132.023	87.93	7.97	d__Bacteria;p__Proteobacteria;c__Gammaproteobacteria;o__SAR86;f__SAR86;g__GCA-2707915;s__
pico132_pico132.046	84	1.72	d__Archaea;p__Thermoplasmatota;c__Poseidoniia;o__Poseidoniales;f__Poseidoniaceae;g__MGIIa-L1;s__MGIIa-L1 sp002506275
pico132_pico132.049	85.91	1.79	d__Bacteria;p__Proteobacteria;c__Gammaproteobacteria;o__Pseudomonadales;f__Pseudohongiellaceae;g__UBA9145;s__UBA9145 sp003483155
pico132_pico132.21	85.42	1.16	d__Bacteria;p__Bacteroidota;c__Bacteroidia;o__Flavobacteriales;f__Flavobacteriaceae;g__;s__
pico132_pico132.3	85.63	1.08	d__Bacteria;p__Proteobacteria;c__Alphaproteobacteria;o__TMED109;f__TMED109;g__GCA-2684605;s__GCA-2684605 sp002684605
pico132_pico132.47	77.3	0.33	d__Bacteria;p__Bacteroidota;c__Bacteroidia;o__Flavobacteriales;f__Flavobacteriaceae;g__MAG-120531;s__
pico132_pico132.53	83.64	4.45	d__Bacteria;p__Proteobacteria;c__Gammaproteobacteria;o__Burkholderiales;f__SG8-40;g__UBA3031;s__UBA3031 sp003483505
pico132_pico132.55	85.11	1.03	d__Bacteria;p__Bacteroidota;c__Bacteroidia;o__Flavobacteriales;f__Schleiferiaceae;g__UBA10364;s__UBA10364 sp003045825
pico132_pico132.58	84.38	1.97	d__Bacteria;p__Proteobacteria;c__Gammaproteobacteria;o__Burkholderiales;f__Methylophilaceae;g__BACL14;s__
pico132_pico132.76	93.1	8.82	d__Bacteria;p__Bacteroidota;c__Bacteroidia;o__Flavobacteriales;f__Flavobacteriaceae;g__MS024-2A;s__
pico132_pico132.80	94.9	0.8	d__Bacteria;p__Proteobacteria;c__Alphaproteobacteria;o__Sphingomonadales;f__Sphingomonadaceae;g__Sphingobium;s__Sphingobium yanoikuyae
pico132_pico132.86	80.73	2.15	d__Bacteria;p__Bacteroidota;c__Bacteroidia;o__Flavobacteriales;f__Schleiferiaceae;g__TMED14;s__
pico132_pico132.91	82.4	4.72	d__Archaea;p__Thermoplasmatota;c__Poseidoniia;o__Poseidoniales;f__Thalassarchaeaceae;g__MGIIb-O2;s__
pico244_pico244.021	86.37	4.42	d__Bacteria;p__Proteobacteria;c__Alphaproteobacteria;o__Rhodobacterales;f__Rhodobacteraceae;g__HIMB11;s__HIMB11 sp003486095
pico244_pico244.22	79.84	2.69	d__Bacteria;p__Bacteroidota;c__Bacteroidia;o__Flavobacteriales;f__Schleiferiaceae;g__UBA10364;s__UBA10364 sp003023665
pico244_pico244.27	76.61	7.04	d__Bacteria;p__Bacteroidota;c__Bacteroidia;o__Flavobacteriales;f__Salibacteraceae;g__SHAN690;s__
pico244_pico244.28	87.78	1.98	d__Bacteria;p__Proteobacteria;c__Gammaproteobacteria;o__Pseudomonadales;f__Pseudohongiellaceae;g__UBA9145;s__
pico244_pico244.31	93.49	2.69	d__Bacteria;p__Planctomycetota;c__UBA8108;o__UBA1146;f__UBA1146;g__UBA12191;s__

pico245_pico245.104	77.54	2.61	d__Bacteria;p__Bacteroidota;c__Bacteroidia;o__Flavobacteriales;f__Schleiferiaceae;g__;s__
pico245_pico245.20	92.75	2.67	d__Bacteria;p__Cyanobacteria;c__Cyanobacteriia;o__PCC-6307;f__Cyanobiaceae;g__Synechococcus_C;s__
pico245_pico245.24	77.3	1.06	d__Bacteria;p__Bacteroidota;c__Bacteroidia;o__Flavobacteriales;f__Flavobacteriaceae;g__Winogradskyella;s__Winogradskyella sp003335675
pico245_pico245.34	87.77	0.57	d__Bacteria;p__Bacteroidota;c__Bacteroidia;o__Flavobacteriales;f__Flavobacteriaceae;g__BACL21;s__
pico245_pico245.36	84.05	2.47	d__Bacteria;p__Bacteroidota;c__Bacteroidia;o__Flavobacteriales;f__Schleiferiaceae;g__UBA10364;s__UBA10364 sp003023665
pico245_pico245.46	84.02	5.9	d__Bacteria;p__Proteobacteria;c__Gammaproteobacteria;o__Pseudomonadales;f__Litoricolaceae;g__Litoricola;s__Litoricola sp002691485
pico245_pico245.8	82.36	1.02	d__Bacteria;p__Proteobacteria;c__Gammaproteobacteria;o__Pseudomonadales;f__Pseudohongiellaceae;g__UBA9145;s__
pico247_pico247.26	88.88	2.9	d__Bacteria;p__Bacteroidota;c__Bacteroidia;o__Flavobacteriales;f__Flavobacteriaceae;g__Polaribacter;s__
pico247_pico247.32	83.12	1.08	d__Bacteria;p__Bacteroidota;c__Bacteroidia;o__Flavobacteriales;f__Schleiferiaceae;g__UBA10364;s__UBA10364 sp003023665
pico247_pico247.38	89.76	2.07	d__Bacteria;p__Proteobacteria;c__Gammaproteobacteria;o__Methylococcales;f__Cycloclasticaceae;g__Cycloclasticus;s__Cycloclasticus sp002700385
pico247_pico247.42	83.72	0.93	d__Bacteria;p__Proteobacteria;c__Gammaproteobacteria;o__Pseudomonadales;f__Pseudohongiellaceae;g__UBA9145;s__
pico247_pico247.55_sub	81.13	1.56	d__Bacteria;p__Proteobacteria;c__Alphaproteobacteria;o__Puniceispirillales;f__Puniceispirillaceae;g__Puniceispirillum;s__
pico264_pico264.0007	84.38	5.83	d__Bacteria;p__Proteobacteria;c__Gammaproteobacteria;o__Pseudomonadales;f__Pseudohongiellaceae;g__OM182;s__OM182 sp003482475
pico264_pico264.0017	76.49	7.15	d__Bacteria;p__Proteobacteria;c__Gammaproteobacteria;o__Pseudomonadales;f__Pseudohongiellaceae;g__OM182;s__
pico264_pico264.0021	92.46	2.05	d__Bacteria;p__Proteobacteria;c__Gammaproteobacteria;o__Pseudomonadales;f__Litoricolaceae;g__Litoricola;s__Litoricola sp002691485
pico264_pico264.10	81.85	2.05	d__Bacteria;p__Proteobacteria;c__Alphaproteobacteria;o__Rhodobacterales;f__Rhodobacteraceae;g__Amylibacter;s__Amylibacter sp900197625
pico264_pico264.35	94.24	4.37	d__Bacteria;p__Proteobacteria;c__Alphaproteobacteria;o__Puniceispirillales;f__Puniceispirillaceae;g__Puniceispirillum;s__
pico264_pico264.40	92.57	0.7	d__Bacteria;p__Bacteroidota;c__Bacteroidia;o__Flavobacteriales;f__Flavobacteriaceae;g__MAG-120531;s__
pico264_pico264.45	88.73	0.96	d__Bacteria;p__Proteobacteria;c__Alphaproteobacteria;o__Rhodobacterales;f__Rhodobacteraceae;g__Planktomarina;s__Planktomarina temperata
pico264_pico264.5	86.39	1.11	d__Bacteria;p__Proteobacteria;c__Gammaproteobacteria;o__Pseudomonadales;f__Porticoccaceae;g__Porticoccus;s__Porticoccus sp002390525
pico265_pico265.0027	75.02	7.34	d__Archaea;p__Thermoplasmatota;c__Poseidoniia;o__Poseidoniales;f__Thalassarchaeaceae;g__MGIIb-O2;s__MGIIb-O2 sp002498985
pico265_pico265.0031_sub	82.41	7.5	d__Bacteria;p__Proteobacteria;c__Alphaproteobacteria;o__Rhodobacterales;f__Rhodobacteraceae;g__LFER01;s__LFER01 sp001642945
pico265_pico265.033	88.62	7.81	d__Bacteria;p__Bacteroidota;c__Bacteroidia;o__Flavobacteriales;f__UA16;g__UBA8752;s__
pico265_pico265.26	82.47	1.82	d__Bacteria;p__Proteobacteria;c__Alphaproteobacteria;o__Rhodobacterales;f__Rhodobacteraceae;g__Amylibacter;s__Amylibacter sp900197625
pico265_pico265.33	90.21	1.23	d__Bacteria;p__Proteobacteria;c__Gammaproteobacteria;o__Pseudomonadales;f__Litoricolaceae;g__Litoricola;s__Litoricola sp002691485
pico265_pico265.47	96.2	0.9	d__Bacteria;p__Proteobacteria;c__Alphaproteobacteria;o__Rhodobacterales;f__Rhodobacteraceae;g__Planktomarina;s__Planktomarina temperata
pico265_pico265.51	84.95	2.22	d__Bacteria;p__Proteobacteria;c__Gammaproteobacteria;o__Pseudomonadales;f__Porticoccaceae;g__Porticoccus;s__Porticoccus sp002390525
pico266_pico266.012	93.2	6.24	d__Bacteria;p__Bacteroidota;c__Bacteroidia;o__Flavobacteriales;f__Schleiferiaceae;g__UBA10364;s__
pico266_pico266.021_sub	87.71	4.82	d__Bacteria;p__Bacteroidota;c__Bacteroidia;o__Flavobacteriales;f__Schleiferiaceae;g__UBA10364;s__UBA10364 sp003045825

pico266_pico266.106	77.45	3.64	d__Bacteria;p__Verrucomicrobiota;c__Verrucomicrobiae;o__Opitutales;f__Opitutaceae;g__UBA5691;s__
pico266_pico266.12	92.85	7.13	d__Bacteria;p__Proteobacteria;c__Alphaproteobacteria;o__Puniceispirillales;f__Puniceispirillaceae;g__Puniceispirillum;s__
pico266_pico266.18	79.44	2.38	d__Bacteria;p__Proteobacteria;c__Alphaproteobacteria;o__Rhodobacterales;f__Rhodobacteraceae;g__Amylibacter;s__Amylibacter sp900197625
pico266_pico266.21	92.39	0.22	d__Bacteria;p__Bacteroidota;c__Bacteroidia;o__Flavobacteriales;f__Flavobacteriaceae;g__MAG-120531;s__
pico266_pico266.24	93.89	0.35	d__Bacteria;p__Proteobacteria;c__Alphaproteobacteria;o__Rhodobacterales;f__Rhodobacteraceae;g__Planktomarina;s__Planktomarina temperata
pico266_pico266.27	75.88	2.22	d__Bacteria;p__Proteobacteria;c__Gammaproteobacteria;o__Pseudomonadales;f__Pseudohongiellaceae;g__OM182;s__OM182 sp003482475
pico266_pico266.28	94.49	0.76	d__Bacteria;p__Bacteroidota;c__Bacteroidia;o__Flavobacteriales;f__Flavobacteriaceae;g__MS024-2A;s__
pico266_pico266.29	91.47	0.56	d__Bacteria;p__Proteobacteria;c__Gammaproteobacteria;o__Pseudomonadales;f__Litoricolaceae;g__Litoricola;s__Litoricola sp002691485
pico266_pico266.3	88.18	5.53	d__Bacteria;p__Proteobacteria;c__Gammaproteobacteria;o__Pseudomonadales;f__Porticoccaceae;g__Porticoccus;s__Porticoccus sp002390525
pico266_pico266.38	85.84	3.41	d__Bacteria;p__Proteobacteria;c__Gammaproteobacteria;o__Burkholderiales;f__Methylophilaceae;g__BACL14;s__
pico266_pico266.6	83.79	0.95	d__Bacteria;p__Bacteroidota;c__Bacteroidia;o__Flavobacteriales;f__Flavobacteriaceae;g__UBA7446;s__
pico281_pico281.012	94.59	3.85	d__Bacteria;p__Actinobacteriota;c__Acidimicrobiia;o__Acidimicrobiales;f__Ilumatobacteraceae;g__Casp-actino5;s__
pico281_pico281.019	94.07	3.61	d__Bacteria;p__Proteobacteria;c__Alphaproteobacteria;o__Rhodobacterales;f__Rhodobacteraceae;g__HIMB11;s__HIMB11 sp003486095
pico281_pico281.021	96.24	8.03	d__Bacteria;p__Proteobacteria;c__Alphaproteobacteria;o__Puniceispirillales;f__Puniceispirillaceae;g__Puniceispirillum;s__
pico281_pico281.039	93.77	2.75	d__Bacteria;p__Proteobacteria;c__Alphaproteobacteria;o__Sphingomonadales;f__Kordiimonadaceae;g__;s__
pico281_pico281.049	98.9	2.09	d__Bacteria;p__Bacteroidota;c__Bacteroidia;o__Flavobacteriales;f__Flavobacteriaceae;g__UBA3478;s__UBA3478 sp003045935
pico281_pico281.066_sub	94.36	8.85	d__Bacteria;p__Proteobacteria;c__Gammaproteobacteria;o__Pseudomonadales;f__Pseudohongiellaceae;g__OM182;s__OM182 sp003482475
pico281_pico281.069_sub	75.9	3.61	d__Bacteria;p__Actinobacteriota;c__Actinomycetia;o__Actinomycetales;f__Microbacteriaceae;g__Pontimonas;s__
pico281_pico281.072	83.86	7.01	d__Bacteria;p__Actinobacteriota;c__Acidimicrobiia;o__Acidimicrobiales;f__Ilumatobacteraceae;g__Casp-actino5;s__
pico281_pico281.22_sub	87.42	0.43	d__Bacteria;p__Bacteroidota;c__Bacteroidia;o__Flavobacteriales;f__Schleiferiaceae;g__UBA10364;s__UBA10364 sp003045825
pico281_pico281.42	89	0.93	d__Bacteria;p__Proteobacteria;c__Gammaproteobacteria;o__Pseudomonadales;f__Litoricolaceae;g__Litoricola;s__Litoricola sp002691485
pico281_pico281.43	91.08	20.2	d__Bacteria;p__Cyanobacteria;c__Cyanobacteriia;o__PCC-6307;f__Cyanobiaceae;g__Cyanobium_A;s__
pico281_pico281.48_sub	89.74	2.13	d__Bacteria;p__Proteobacteria;c__Alphaproteobacteria;o__Rhodobacterales;f__Rhodobacteraceae;g__Planktomarina;s__Planktomarina temperata
pico281_pico281.5	97.61	0.84	d__Bacteria;p__Bacteroidota;c__Bacteroidia;o__Flavobacteriales;f__Flavobacteriaceae;g__Kordia;s__
pico281_pico281.58	84.6	2.07	d__Bacteria;p__Proteobacteria;c__Gammaproteobacteria;o__Pseudomonadales;f__HTCC2089;g__;s__
pico281_pico281.6	77.82	3.06	d__Bacteria;p__Actinobacteriota;c__Actinomycetia;o__Nanopelagicales;f__S36-B12;g__Mxb001;s__
pico281_pico281.7	84.48	5.96	d__Bacteria;p__Cyanobacteria;c__Cyanobacteriia;o__PCC-6307;f__Cyanobiaceae;g__Synechococcus_E;s__
pico281_pico281.8	97.94	2.15	d__Bacteria;p__Bacteroidota;c__Bacteroidia;o__Flavobacteriales;f__UBA10329;g__UBA10329;s__
pico282_pico282.004	93.38	3.08	d__Bacteria;p__Proteobacteria;c__Alphaproteobacteria;o__Rhodobacterales;f__Rhodobacteraceae;g__HIMB11;s__HIMB11 sp003486095

pico282_pico282.006	89.5	8.31	d__Bacteria;p__Proteobacteria;c__Gammaproteobacteria;o__Pseudomonadales;f__Halieaceae;g__Luminiphilus;s__Luminip hilus sp002691565
pico282_pico282.020_sub	80	7.51	d__Bacteria;p__Proteobacteria;c__Gammaproteobacteria;o__Burkholderiales;f__Methylophilaceae;g__BACL14;s__
pico282_pico282.025_sub	76.36	7.79	d__Bacteria;p__Proteobacteria;c__Gammaproteobacteria;o__Pseudomonadales;f__Halieaceae;g__Luminiphilus;s__
pico282_pico282.20	87.77	2.32	d__Bacteria;p__Bacteroidota;c__Bacteroidia;o__Flavobacteriales;f__Flavobacteriaceae;g__UBA3478;s__
pico282_pico282.22	89.71	1.37	d__Bacteria;p__Proteobacteria;c__Alphaproteobacteria;o__Rhodobacterales;f__Rhodobacteraceae;g__Planktomarina;s__Pl anktomarina temperata
pico282_pico282.23	87.23	3.72	d__Bacteria;p__Verrucomicrobiota;c__Verrucomicrobiae;o__Opitutales;f__Puniceicoccaceae;g__;s__
pico282_pico282.27	83.35	2.47	d__Bacteria;p__Proteobacteria;c__Alphaproteobacteria;o__Rhodobacterales;f__Rhodobacteraceae;g__MED-G52;s__MED-G52 sp002457055
pico282_pico282.34	76.82	5.12	d__Bacteria;p__Proteobacteria;c__Alphaproteobacteria;o__Rhodobacterales;f__Rhodobacteraceae;g__Amylibacter;s__Amyl ibacter sp900197625
pico282_pico282.37	90.13	5.07	d__Bacteria;p__Proteobacteria;c__Gammaproteobacteria;o__Pseudomonadales;f__Halieaceae;g__Luminiphilus;s__
pico282_pico282.44	98.53	0.83	d__Bacteria;p__Bacteroidota;c__Bacteroidia;o__Flavobacteriales;f__Flavobacteriaceae;g__UBA3478;s__UBA3478 sp003045935
pico282_pico282.45	81.33	0.13	d__Archaea;p__Thermoplasmatota;c__Poseidoniia;o__Poseidoniales;f__Poseidoniaceae;g__MGIIa-K1;s__
pico282_pico282.49	83.19	0.93	d__Bacteria;p__Proteobacteria;c__Gammaproteobacteria;o__Pseudomonadales;f__Porticoccaceae;g__HTCC2207;s__
pico282_pico282.53	83.12	6.68	d__Bacteria;p__Bacteroidota;c__Bacteroidia;o__Flavobacteriales;f__Flavobacteriaceae;g__Winogradskyella;s__Winogradsk yella sp003335675
pico282_pico282.54	83.35	0.49	d__Bacteria;p__Proteobacteria;c__Gammaproteobacteria;o__Pseudomonadales;f__Litoricolaceae;g__Litoricola;s__Litoricola sp002691485
pico282_pico282.56	91.3	0.54	d__Bacteria;p__Bacteroidota;c__Bacteroidia;o__Flavobacteriales;f__Schleiferiaceae;g__UBA10364;s__UBA10364 sp003023665
pico282_pico282.57	89.14	1.67	d__Bacteria;p__Proteobacteria;c__Gammaproteobacteria;o__Pseudomonadales;f__UBA7434;g__UBA7434;s__UBA7434 sp002480045
pico282_pico282.69	86.83	2.58	d__Bacteria;p__Proteobacteria;c__Alphaproteobacteria;o__Puniceispirillales;f__Puniceispirillaceae;g__Puniceispirillum;s__
pico284_pico284.003	92.84	7.42	d__Bacteria;p__Proteobacteria;c__Gammaproteobacteria;o__Pseudomonadales;f__Porticoccaceae;g__HTCC2207;s__
pico284_pico284.010_sub	89.04	6.48	d__Bacteria;p__Proteobacteria;c__Alphaproteobacteria;o__Rhodobacterales;f__Rhodobacteraceae;g__HIMB11;s__HIMB11 sp003486095
pico284_pico284.016_sub	91.02	1.3	d__Bacteria;p__Proteobacteria;c__Gammaproteobacteria;o__Pseudomonadales;f__Litoricolaceae;g__Litoricola;s__Litoricola sp002691485
pico284_pico284.018_sub	82.19	0.85	d__Bacteria;p__Actinobacteriota;c__Acidimicrobiia;o__Acidimicrobiales;f__Ilumatobacteraceae;g__Casp-actino5;s__
pico284_pico284.039_sub	95.99	2.53	d__Bacteria;p__Proteobacteria;c__Alphaproteobacteria;o__Puniceispirillales;f__Puniceispirillaceae;g__Puniceispirillum;s__
pico284_pico284.124	76.07	2.94	d__Bacteria;p__Planctomycetota;c__UBA8742;o__UBA2392;f__;g__;s__
pico284_pico284.22	88.43	3.02	d__Bacteria;p__Proteobacteria;c__Alphaproteobacteria;o__Puniceispirillales;f__Puniceispirillaceae;g__Puniceispirillum;s__
pico284_pico284.26	82.94	1.4	d__Bacteria;p__Proteobacteria;c__Alphaproteobacteria;o__Rhodobacterales;f__Rhodobacteraceae;g__LFER01;s__
pico284_pico284.28	84.73	1.63	d__Bacteria;p__Bacteroidota;c__Bacteroidia;o__Flavobacteriales;f__Schleiferiaceae;g__UBA10364;s__UBA10364 sp003023665
pico284_pico284.3	94.42	2.42	d__Bacteria;p__Proteobacteria;c__Alphaproteobacteria;o__Parvibaculales;f__RS24;g__UBA8337;s__UBA8337 sp900197605
pico284_pico284.33	79.31	4.35	d__Bacteria;p__Cyanobacteria;c__Cyanobacteriia;o__PCC-6307;f__Cyanobiaceae;g__Synechococcus_E;s__
pico284_pico284.35	81.59	0.92	d__Bacteria;p__Planctomycetota;c__Phycisphaerae;o__Phycisphaerales;f__Phycisphaeraceae;g__;s__

pico284_pico284.38	92.71	6.96	d__Bacteria;p__Proteobacteria;c__Alphaproteobacteria;o__Parvibaculales;f__RS24;g__UBA8337;s__
pico284_pico284.39	93.71	1.44	d__Bacteria;p__Verrucomicrobiota;c__Verrucomicrobiae;o__Verrucomicrobiales;f__Akkermansiaceae;g__SW10;s__
pico284_pico284.42	91.67	0.68	d__Bacteria;p__Bacteroidota;c__Bacteroidia;o__Flavobacteriales;f__Flavobacteriaceae;g__Kordia;s__
pico284_pico284.45	75.81	3.76	d__Bacteria;p__Bacteroidota;c__Bacteroidia;o__Flavobacteriales;f__BACL11;g__UBA8444;s__
pico284_pico284.66	93.16	1.71	d__Bacteria;p__Actinobacteriota;c__Acidimicrobiia;o__Acidimicrobiales;f__UBA11606;g__UBA11606;s__
pico284_pico284.84	82.91	0.95	d__Bacteria;p__Actinobacteriota;c__Acidimicrobiia;o__Acidimicrobiales;f__Ilumatobacteraceae;g__Casp-actino8;s__
pico304_pico304.021	94.06	4.23	d__Bacteria;p__Proteobacteria;c__Gammaproteobacteria;o__Pseudomonadales;f__Litoricolaceae;g__Litoricola;s__Litoricola sp002691485
pico304_pico304.038	87.9	5.66	d__Bacteria;p__Bacteroidota;c__Bacteroidia;o__Flavobacteriales;f__Schleiferiaceae;g__;s__
pico304_pico304.21	75.39	1.24	d__Bacteria;p__Bacteroidota;c__Bacteroidia;o__Flavobacteriales;f__Flavobacteriaceae;g__Winogradskyella;s__
pico304_pico304.24	82.79	2.21	d__Bacteria;p__Proteobacteria;c__Gammaproteobacteria;o__Pseudomonadales;f__Pseudohongiellaceae;g__UBA9145;s__
pico304_pico304.46	86.13	1.6	d__Bacteria;p__Actinobacteriota;c__Acidimicrobiia;o__Acidimicrobiales;f__Ilumatobacteraceae;g__Casp-actino5;s__
pico497_pico497.020_sub	89.77	8.4	d__Bacteria;p__Bacteroidota;c__Bacteroidia;o__Flavobacteriales;f__Flavobacteriaceae;g__CAU-1491;s__
pico497_pico497.11	84.48	7.34	d__Bacteria;p__Proteobacteria;c__Gammaproteobacteria;o__Pseudomonadales;f__Halieaceae;g__Luminiphilus;s__Luminiphilus sp002390485
pico497_pico497.113	79.72	14.4	d__Bacteria;p__Verrucomicrobiota;c__Verrucomicrobiae;o__Verrucomicrobiales;f__Akkermansiaceae;g__UBA985;s__
pico497_pico497.23	84.76	0.14	d__Bacteria;p__Bacteroidota;c__Bacteroidia;o__Flavobacteriales;f__Flavobacteriaceae;g__MAG-120531;s__
pico497_pico497.24	90.86	0.43	d__Bacteria;p__Proteobacteria;c__Gammaproteobacteria;o__Pseudomonadales;f__Porticoccaceae;g__HTCC2207;s__HTCC2207 sp002457245
pico497_pico497.29	95.47	0.87	d__Bacteria;p__Proteobacteria;c__Alphaproteobacteria;o__Puniceispirillales;f__Puniceispirillaceae;g__Puniceispirillum;s__
pico497_pico497.30	79.3	3.08	d__Bacteria;p__Proteobacteria;c__Alphaproteobacteria;o__Caulobacterales;f__Maricaulaceae;g__Hellea;s__
pico497_pico497.32	95.22	1.1	d__Bacteria;p__Bacteroidota;c__Bacteroidia;o__Flavobacteriales;f__Flavobacteriaceae;g__MS024-2A;s__
pico497_pico497.34	81.81	5.33	d__Bacteria;p__Proteobacteria;c__Gammaproteobacteria;o__Pseudomonadales;f__HTCC2089;g__UBA4582;s__UBA4582 sp002389265
pico497_pico497.38	88.26	2.11	d__Bacteria;p__Proteobacteria;c__Alphaproteobacteria;o__Rhodobacterales;f__Rhodobacteraceae;g__LFER01;s__LFER01 sp001642945
pico497_pico497.41	85.45	1.04	d__Bacteria;p__Bacteroidota;c__Bacteroidia;o__Flavobacteriales;f__Flavobacteriaceae;g__UBA3537;s__UBA3537 sp001735715
pico497_pico497.43	90.17	0.42	d__Bacteria;p__Proteobacteria;c__Gammaproteobacteria;o__Pseudomonadales;f__Litoricolaceae;g__Litoricola;s__Litoricola sp002691485
pico497_pico497.44	90.65	3.33	d__Bacteria;p__Proteobacteria;c__Gammaproteobacteria;o__Pseudomonadales;f__HTCC2089;g__UBA9926;s__
pico497_pico497.45	91.11	1.53	d__Bacteria;p__Proteobacteria;c__Gammaproteobacteria;o__Pseudomonadales;f__HTCC2089;g__UBA4421;s__
pico497_pico497.50	78.48	1.75	d__Bacteria;p__Proteobacteria;c__Gammaproteobacteria;o__Burkholderiales;f__Methylophilaceae;g__BACL14;s__
pico497_pico497.58	80.54	1.29	d__Bacteria;p__Proteobacteria;c__Alphaproteobacteria;o__Rhodobacterales;f__Rhodobacteraceae;g__Amylibacter;s__Amylibacter sp900197625
pico497_pico497.6	94.47	0.48	d__Bacteria;p__Proteobacteria;c__Alphaproteobacteria;o__Rhodobacterales;f__Rhodobacteraceae;g__Planktomarina;s__Planktomarina temperata
pico497_pico497.62	83.76	1.83	d__Bacteria;p__Proteobacteria;c__Gammaproteobacteria;o__Burkholderiales;f__SG8-40;g__UBA3031;s__UBA3031 sp003485335

pico539_pico539.006	96.37	8.93	d__Bacteria;p__Proteobacteria;c__Gammaproteobacteria;o__Pseudomonadales;f__Litoricolaceae;g__Litoricola;s__Litoricola sp002691485
pico539_pico539.009	90.51	3.66	d__Bacteria;p__Proteobacteria;c__Gammaproteobacteria;o__Pseudomonadales;f__Halieaceae;g__Luminiphilus;s__Luminip hilus sp002691565
pico539_pico539.16	84.29	1.59	d__Bacteria;p__Proteobacteria;c__Gammaproteobacteria;o__Methylococcales;f__Cycloclasticaceae;g__Cycloclasticus;s__C ycloclasticus pugetii
pico539_pico539.2_sub	81.13	0.54	d__Bacteria;p__Proteobacteria;c__Alphaproteobacteria;o__Rhodobacterales;f__Rhodobacteraceae;g__Planktomarina;s__Pl anktomarina temperata
pico539_pico539.23	89.97	1.75	d__Bacteria;p__Bacteroidota;c__Bacteroidia;o__Flavobacteriales;f__Schleiferiaceae;g__UBA10364;s__UBA10364 sp003023665
pico539_pico539.35	91.26	1.92	d__Bacteria;p__Proteobacteria;c__Alphaproteobacteria;o__Rhodobacterales;f__Rhodobacteraceae;g__HIMB11;s__HIMB11 sp003486095
pico539_pico539.8	89.68	1.47	d__Bacteria;p__Bacteroidota;c__Bacteroidia;o__Flavobacteriales;f__Flavobacteriaceae;g__UBA3478;s__UBA3478 sp003045935
pico540_pico540.003	91.46	3.09	d__Bacteria;p__Proteobacteria;c__Alphaproteobacteria;o__Rhodobacterales;f__Rhodobacteraceae;g__HIMB11;s__HIMB11 sp003486095
pico540_pico540.010	96.02	6.44	d__Bacteria;p__Verrucomicrobiota;c__Verrucomicrobiae;o__Opitutales;f__Puniceicoccaceae;g__;s__
pico540_pico540.012	87.71	4.7	d__Bacteria;p__Proteobacteria;c__Gammaproteobacteria;o__Pseudomonadales;f__Halieaceae;g__Luminiphilus;s__Luminip hilus sp002691565
pico540_pico540.027_sub	87.68	6.04	d__Bacteria;p__Bacteroidota;c__Bacteroidia;o__Flavobacteriales;f__Schleiferiaceae;g__UBA10364;s__
pico540_pico540.18	84.84	2.43	d__Bacteria;p__Bacteroidota;c__Bacteroidia;o__Flavobacteriales;f__Flavobacteriaceae;g__UBA3478;s__
pico540_pico540.27	93.26	1.6	d__Bacteria;p__Proteobacteria;c__Gammaproteobacteria;o__Pseudomonadales;f__Litoricolaceae;g__Litoricola;s__Litoricola sp002691485
pico540_pico540.28	83.87	0.54	d__Bacteria;p__Bacteroidota;c__Bacteroidia;o__Flavobacteriales;f__Schleiferiaceae;g__UBA10364;s__UBA10364 sp003023665
pico540_pico540.38	75.54	0.77	d__Bacteria;p__Bacteroidota;c__Bacteroidia;o__Flavobacteriales;f__Flavobacteriaceae;g__UBA3478;s__UBA3478 sp003045935
pico540_pico540.5	87.76	1.62	d__Bacteria;p__Proteobacteria;c__Alphaproteobacteria;o__Puniceispirillales;f__Puniceispirillaceae;g__;s__
pico550_pico550.028	95.79	3.04	d__Bacteria;p__Proteobacteria;c__Gammaproteobacteria;o__Pseudomonadales;f__Litoricolaceae;g__Litoricola;s__Litoricola sp002691485
pico550_pico550.037	87.41	9.97	d__Bacteria;p__Cyanobacteria;c__Cyanobacteriia;o__PCC-6307;f__Cyanobiaceae;g__Vulcanococcus;s__Vulcanococcus sp000179255
pico550_pico550.14	84.38	3.39	d__Bacteria;p__Bacteroidota;c__Bacteroidia;o__Flavobacteriales;f__Flavobacteriaceae;g__Winogradskyella;s__Winogradsk yella sp003335675
pico550_pico550.28	82.65	1.48	d__Bacteria;p__Proteobacteria;c__Gammaproteobacteria;o__Pseudomonadales;f__Pseudohongiellaceae;g__UBA9145;s__
pico550_pico550.45	82.71	1.66	d__Bacteria;p__Bacteroidota;c__Bacteroidia;o__Flavobacteriales;f__Schleiferiaceae;g__UBA10364;s__UBA10364 sp003023665
pico550_pico550.49	88.94	2.41	d__Bacteria;p__Bacteroidota;c__Bacteroidia;o__Flavobacteriales;f__Flavobacteriaceae;g__UBA3478;s__
pico551_pico551.013	89.74	4.2	d__Bacteria;p__Proteobacteria;c__Gammaproteobacteria;o__Pseudomonadales;f__Litoricolaceae;g__Litoricola;s__Litoricola sp002691485
pico551_pico551.11	84.34	1.46	d__Bacteria;p__Proteobacteria;c__Alphaproteobacteria;o__Rhodobacterales;f__Rhodobacteraceae;g__LFER01;s__
pico551_pico551.14	85.16	1.79	d__Bacteria;p__Proteobacteria;c__Gammaproteobacteria;o__Pseudomonadales;f__Pseudohongiellaceae;g__UBA9145;s__
pico551_pico551.4	82.35	4.85	d__Bacteria;p__Bacteroidota;c__Bacteroidia;o__Flavobacteriales;f__Flavobacteriaceae;g__Winogradskyella;s__Winogradsk yella sp003335675
pico552_pico552.021	90	2.59	d__Bacteria;p__Proteobacteria;c__Gammaproteobacteria;o__Pseudomonadales;f__Pseudohongiellaceae;g__UBA9145;s__
pico552_pico552.026	91.23	9.3	d__Bacteria;p__Proteobacteria;c__Gammaproteobacteria;o__Pseudomonadales;f__Litoricolaceae;g__Litoricola;s__Litoricola sp002691485

Table S4. Proportional Similarity index for all high quality, dereplicated MAGs used in the study. Main type and subtype of PS index are calculated according to the equation shown in the Material and Methods section.

	PS_maintype	Rank_transformed_PS_maintype	PS_subtype	Rank_transformed_PS_subtype	Rank_product	g_s
pico127_pico127.002	0.423458459	56	0.42345846	69	3864	specialists
pico127_pico127.018	0.388136546	36	0.26391317	26	936	specialists
pico127_pico127.023	0.368573156	19	0.21413539	22	418	specialists
pico127_pico127.035	0.63576366	126	0.52752312	115	14490	potential_specialists
pico127_pico127.045	0.368421053	10	0.36842105	47	470	specialists
pico127_pico127.062	0.431132576	64	0.3110947	31	1984	specialists
pico127_pico127.12	0.368421053	11	0.15789474	2	22	specialists
pico127_pico127.14	0.374401623	30	0.19480945	15	450	specialists
pico127_pico127.15	0.396249494	41	0.18572318	13	533	specialists
pico127_pico127.16	0.452641622	74	0.45264162	86	6364	potential_specialists
pico127_pico127.19	0.369623126	21	0.2138458	21	441	specialists
pico127_pico127.25	0.562866455	106	0.51621983	109	11554	potential_specialists
pico127_pico127.37	0.425467381	58	0.42546738	71	4118	potential_specialists
pico127_pico127.47_sub	0.422113545	55	0.36605702	46	2530	specialists
pico127_pico127.51	0.370762565	26	0.35848922	45	1170	specialists
pico127_pico127.64	0.379665689	32	0.22604431	25	800	specialists
pico127_pico127.69	0.473121423	83	0.33280089	39	3237	specialists
pico131_pico131.002	0.436110027	65	0.43611003	77	5005	potential_specialists
pico131_pico131.013_sub	0.420230952	53	0.31395254	32	1696	specialists
pico131_pico131.022	0.561159425	104	0.56115943	123	12792	potential_specialists
pico131_pico131.024	0.507118015	94	0.50711802	108	10152	potential_specialists
pico131_pico131.047	0.379613634	31	0.27880432	28	868	specialists
pico131_pico131.048	0.569082781	108	0.43072985	76	8208	potential_specialists
pico131_pico131.067	0.53950108	99	0.53950108	117	11583	potential_specialists

pico131_pico131.1	0.368421053	12	0.15789474	3	36	specialists
pico131_pico131.11	0.399710489	44	0.39971049	60	2640	specialists
pico131_pico131.18	0.374148902	29	0.19081107	14	406	specialists
pico131_pico131.28	0.477135272	87	0.47713527	103	8961	potential_specialists
pico131_pico131.30	0.369948435	23	0.2088105	19	437	specialists
pico131_pico131.50	0.369439545	20	0.17008161	9	180	specialists
pico131_pico131.52	0.650916648	135	0.58219334	128	17280	potential_specialists
pico131_pico131.78	0.501790492	91	0.42672819	73	6643	potential_specialists
pico131_pico131.84	0.400183247	45	0.40018325	61	2745	specialists
pico132_pico132.003	0.430605081	62	0.43060508	74	4588	potential_specialists
pico132_pico132.014	0.557862425	102	0.55786243	121	12342	potential_specialists
pico132_pico132.019	0.370491173	24	0.17545465	12	288	specialists
pico132_pico132.023	0.37049483	25	0.17157076	10	250	specialists
pico132_pico132.046	0.373272922	28	0.21114827	20	560	specialists
pico132_pico132.049	0.372330492	27	0.35660097	44	1188	specialists
pico132_pico132.21	0.369896297	22	0.19913859	16	352	specialists
pico132_pico132.47	0.475200469	85	0.47520047	100	8500	potential_specialists
pico132_pico132.53	0.631578947	120	0.57501467	126	15120	potential_specialists
pico132_pico132.55	0.531840462	97	0.53184046	116	11252	potential_specialists
pico132_pico132.58	0.400614931	46	0.40061493	62	2852	specialists
pico132_pico132.76	0.410929366	50	0.41092937	65	3250	specialists
pico132_pico132.80	0.368421053	13	0.15789474	4	52	specialists
pico132_pico132.86	0.368421053	14	0.15789474	5	70	specialists
pico132_pico132.91	0.368421053	15	0.15789474	6	90	specialists
pico244_pico244.021	0.583159757	111	0.58315976	130	14430	potential_specialists
pico244_pico244.22	0.877163298	190	0.81938119	192	36480	generalists
pico244_pico244.27	0.822616536	180	0.54485225	119	21420	generalists

pico244_pico244.28	0.736842105	151	0.52308377	112	16912	potential_specialists
pico244_pico244.31	0.368421053	16	0.31578947	33	528	specialists
pico245_pico245.104	0.729576935	150	0.72957694	169	25350	generalists
pico245_pico245.20	0.399304254	43	0.39930425	59	2537	specialists
pico245_pico245.24	0.848327247	184	0.80004032	184	33856	generalists
pico245_pico245.34	0.411401648	51	0.41140165	66	3366	specialists
pico245_pico245.36	0.785388171	164	0.73923476	171	28044	generalists
pico245_pico245.46	0.873322775	189	0.87332278	196	37044	generalists
pico245_pico245.8	0.368421053	17	0.36842105	48	816	specialists
pico247_pico247.26	0.430238065	61	0.32984125	38	2318	specialists
pico247_pico247.32	0.786770663	165	0.74447828	172	28380	generalists
pico247_pico247.38	0.708718616	148	0.60702685	138	20424	generalists
pico247_pico247.42	0.736842105	152	0.5169276	111	16872	potential_specialists
pico247_pico247.55_sub	0.859885256	186	0.64950016	148	27528	generalists
pico264_pico264.0007	0.583828953	112	0.58382895	132	14784	potential_specialists
pico264_pico264.0017	0.631578947	116	0.54933814	120	13920	potential_specialists
pico264_pico264.0021	0.742954057	156	0.69973034	163	25428	generalists
pico264_pico264.10	0.424617986	57	0.42461799	70	3990	specialists
pico264_pico264.35	0.690546661	147	0.65778919	151	22197	generalists
pico264_pico264.40	0.453082912	75	0.45308291	87	6525	potential_specialists
pico264_pico264.45	0.444382305	70	0.44438231	81	5670	potential_specialists
pico264_pico264.5	0.390515182	38	0.39051518	54	2052	specialists
pico265_pico265.0027	0.38236677	33	0.33500781	40	1320	specialists
pico265_pico265.0031_sub	0.552413487	101	0.45518174	90	9090	potential_specialists
pico265_pico265.033	0.85593742	185	0.71532754	166	30710	generalists
pico265_pico265.26	0.446349242	71	0.44634924	82	5822	potential_specialists
pico265_pico265.33	0.80560775	175	0.80560775	186	32550	generalists

pico265_pico265.47	0.443322504	69	0.4433225	80	5520	potential_specialists
pico265_pico265.51	0.39330795	39	0.39330795	55	2145	specialists
pico266_pico266.012	0.497613626	90	0.39715618	58	5220	potential_specialists
pico266_pico266.021_sub	0.51642883	95	0.51642883	110	10450	potential_specialists
pico266_pico266.106	0.477251097	88	0.46489989	95	8360	potential_specialists
pico266_pico266.12	0.744500514	157	0.58240823	129	20253	generalists
pico266_pico266.18	0.446894707	72	0.44689471	83	5976	potential_specialists
pico266_pico266.21	0.467877787	81	0.46787779	96	7776	potential_specialists
pico266_pico266.24	0.43068744	63	0.43068744	75	4725	potential_specialists
pico266_pico266.27	0.560495195	103	0.5604952	122	12566	potential_specialists
pico266_pico266.28	0.416246309	52	0.41624631	67	3484	specialists
pico266_pico266.29	0.790423224	168	0.79042322	180	30240	generalists
pico266_pico266.3	0.395619079	40	0.39561908	57	2280	specialists
pico266_pico266.38	0.403594818	49	0.40359482	64	3136	specialists
pico266_pico266.6	0.421489444	54	0.3944986	56	3024	specialists
pico281_pico281.012	0.562446661	105	0.45012795	84	8820	potential_specialists
pico281_pico281.019	0.681197855	146	0.68119786	160	23360	generalists
pico281_pico281.021	0.784992584	163	0.58052541	127	20701	generalists
pico281_pico281.039	0.263157895	1	0.15789474	7	7	specialists
pico281_pico281.049	0.649566718	134	0.63816212	145	19430	generalists
pico281_pico281.066_sub	0.637376654	127	0.58376149	131	16637	potential_specialists
pico281_pico281.069_sub	0.661892478	139	0.62372096	142	19738	generalists
pico281_pico281.072	0.803858908	173	0.67049961	155	26815	generalists
pico281_pico281.22_sub	0.631578947	117	0.56390397	124	14508	potential_specialists
pico281_pico281.42	0.796773633	171	0.79677363	183	31293	generalists
pico281_pico281.43	0.451883531	73	0.35607904	43	3139	specialists

pico281_pico281.48_sub	0.453392708	76	0.45339271	88	6688	potential_specialists
pico281_pico281.5	0.263157895	2	0.15789474	8	16	specialists
pico281_pico281.58	0.456705756	78	0.45670576	92	7176	potential_specialists
pico281_pico281.6	0.551484855	100	0.37016243	50	5000	potential_specialists
pico281_pico281.7	0.640098372	130	0.58441921	134	17420	potential_specialists
pico281_pico281.8	0.631578947	118	0.31578947	34	4012	potential_specialists
pico282_pico282.004	0.6403223	131	0.6403223	147	19257	generalists
pico282_pico282.006	0.671593237	142	0.67159324	156	22152	generalists
pico282_pico282.020_sub	0.828916846	182	0.8168228	189	34398	generalists
pico282_pico282.025_sub	0.663334117	140	0.6325504	143	20020	generalists
pico282_pico282.20	0.664713248	141	0.66471325	153	21573	generalists
pico282_pico282.22	0.45686551	79	0.45686551	93	7347	potential_specialists
pico282_pico282.23	0.291370205	6	0.29137021	29	174	specialists
pico282_pico282.27	0.677997507	145	0.67799751	159	23055	generalists
pico282_pico282.34	0.493896425	89	0.49389643	106	9434	potential_specialists
pico282_pico282.37	0.635662941	125	0.45667272	91	11375	potential_specialists
pico282_pico282.44	0.814183258	177	0.69188627	162	28674	generalists
pico282_pico282.45	0.632700831	122	0.5434132	118	14396	potential_specialists
pico282_pico282.49	0.278869412	4	0.22598072	24	96	specialists
pico282_pico282.53	0.871388376	188	0.81458879	188	35344	generalists
pico282_pico282.54	0.777679987	161	0.77461966	176	28336	generalists
pico282_pico282.56	0.909869328	196	0.84599718	194	38024	generalists
pico282_pico282.57	0.573632863	110	0.37437544	51	5610	potential_specialists
pico282_pico282.69	0.585079555	113	0.58507956	135	15255	potential_specialists
pico284_pico284.003	0.271301532	3	0.22113624	23	69	specialists
pico284_pico284.010_sub	0.652653101	137	0.6526531	150	20550	generalists

pico284_pico284.016_sub	0.809456719	176	0.73818279	170	29920	generalists
pico284_pico284.018_sub	0.564506002	107	0.45206288	85	9095	potential_specialists
pico284_pico284.039_sub	0.794761335	170	0.61182428	139	23630	generalists
pico284_pico284.124	0.537085871	98	0.31578947	35	3430	specialists
pico284_pico284.22	0.59071919	114	0.59071919	137	15618	potential_specialists
pico284_pico284.26	0.76364092	160	0.70299222	164	26240	generalists
pico284_pico284.28	0.786840161	166	0.74531885	173	28718	generalists
pico284_pico284.3	0.503421902	93	0.49997918	107	9951	potential_specialists
pico284_pico284.33	0.641764445	132	0.58603094	136	17952	generalists
pico284_pico284.35	0.279677509	5	0.17441435	11	55	specialists
pico284_pico284.38	0.633017633	123	0.48839066	105	12915	potential_specialists
pico284_pico284.39	0.296261077	8	0.27554128	27	216	specialists
pico284_pico284.42	0.403237172	48	0.29797401	30	1440	specialists
pico284_pico284.45	0.76130285	159	0.61406967	140	22260	generalists
pico284_pico284.66	0.29618981	7	0.20305605	17	119	specialists
pico284_pico284.84	0.45740534	80	0.35214218	42	3360	specialists
pico304_pico304.021	0.882871728	191	0.78105113	178	33998	generalists
pico304_pico304.038	0.82235419	179	0.81857435	191	34189	generalists
pico304_pico304.21	0.903779827	194	0.88643037	197	38218	generalists
pico304_pico304.24	0.736842105	154	0.52631579	114	17556	generalists
pico304_pico304.46	0.634059936	124	0.62078295	141	17484	generalists
pico497_pico497.020_sub	0.468014967	82	0.46801497	97	7954	potential_specialists
pico497_pico497.11	0.398377457	42	0.20406253	18	756	specialists
pico497_pico497.113	0.790461853	169	0.75942498	174	29406	generalists
pico497_pico497.23	0.475211368	86	0.47521137	101	8686	potential_specialists
pico497_pico497.24	0.440497975	67	0.14141051	1	67	specialists
pico497_pico497.29	0.777987443	162	0.68858344	161	26082	generalists

pico497_pico497.30	0.425662299	59	0.32651485	37	2183	specialists
pico497_pico497.32	0.390184283	37	0.34259029	41	1517	specialists
pico497_pico497.34	0.368421053	18	0.36842105	49	882	specialists
pico497_pico497.38	0.571407805	109	0.47611046	102	11118	potential_specialists
pico497_pico497.41	0.627042251	115	0.58438548	133	15295	potential_specialists
pico497_pico497.43	0.825211701	181	0.8252117	193	34933	generalists
pico497_pico497.44	0.639132273	128	0.47278698	98	12544	potential_specialists
pico497_pico497.45	0.517694348	96	0.46370467	94	9024	potential_specialists
pico497_pico497.50	0.401854744	47	0.40185474	63	2961	specialists
pico497_pico497.58	0.425943775	60	0.42594378	72	4320	potential_specialists
pico497_pico497.6	0.436242542	66	0.43624254	78	5148	potential_specialists
pico497_pico497.62	0.631710406	121	0.57427787	125	15125	potential_specialists
pico539_pico539.006	0.454219914	77	0.45421991	89	6853	potential_specialists
pico539_pico539.009	0.675638785	144	0.67563879	158	22752	generalists
pico539_pico539.16	0.631578947	119	0.48242034	104	12376	potential_specialists
pico539_pico539.2_sub	0.440732288	68	0.44073229	79	5372	potential_specialists
pico539_pico539.23	0.904921777	195	0.85020243	195	38025	generalists
pico539_pico539.35	0.658872813	138	0.65887281	152	20976	generalists
pico539_pico539.8	0.64862534	133	0.63402808	144	19152	generalists
pico540_pico540.003	0.639564838	129	0.63956484	146	18834	generalists
pico540_pico540.010	0.316447668	9	0.31644767	36	324	specialists
pico540_pico540.012	0.675085314	143	0.67508531	157	22451	generalists
pico540_pico540.027_sub	0.723747451	149	0.72374745	167	24883	generalists
pico540_pico540.18	0.651685597	136	0.6516856	149	20264	generalists
pico540_pico540.27	0.817286762	178	0.81728676	190	33820	generalists
pico540_pico540.28	0.865326377	187	0.80893206	187	34969	generalists
pico540_pico540.38	0.739865506	155	0.72652938	168	26040	generalists

pico540_pico540.5	0.501966447	92	0.42111139	68	6256	potential_specialists
pico550_pico550.028	0.895886411	193	0.79539277	182	35126	generalists
pico550_pico550.037	0.757818404	158	0.66745176	154	24332	generalists
pico550_pico550.14	0.843786929	183	0.80327018	185	33855	generalists
pico550_pico550.28	0.473403806	84	0.47340381	99	8316	potential_specialists
pico550_pico550.45	0.788307111	167	0.77279984	175	29225	generalists
pico550_pico550.49	0.386092589	35	0.38609259	53	1855	specialists
pico551_pico551.013	0.884680099	192	0.78746679	179	34368	generalists
pico551_pico551.11	0.804669956	174	0.70581874	165	28710	generalists
pico551_pico551.14	0.736842105	153	0.52631579	113	17289	potential_specialists
pico551_pico551.4	0.801339111	172	0.77648755	177	30444	generalists
pico552_pico552.021	0.384400725	34	0.38440073	52	1768	specialists
pico552_pico552.026	0.919191039	197	0.7931623	181	35657	generalists

References cited

1. Amaral-Zettler LA, Rocca JD, Lamontagne MG *et al.* Changes in microbial community structure in the wake of hurricanes katrina and rita. *Environmental science & technology*. 2008;**42**:9072-78
2. Mallin MA, Corbett CA. How hurricane attributes determine the extent of environmental effects: Multiple hurricanes and different coastal systems. *Estuaries and Coasts*. 2006;**29**:1046-61
3. Huisman J, Codd GA, Paerl HW *et al.* Cyanobacterial blooms. *Nature Reviews Microbiology*. 2018;**16**:471-83
4. Ward CS, Yung C-M, Davis KM *et al.* Annual community patterns are driven by seasonal switching between closely related marine bacteria. *The ISME Journal*. 2017;**11**:1412-22 https://doi.org/10.1038/ismej.2017.4
5. Edgar RC. Unoise2: Improved error-correction for illumina 16s and its amplicon sequencing. *BioRxiv*. 2016:081257
6. Edgar RC. Sintax: A simple non-bayesian taxonomy classifier for 16s and its sequences. *bioRxiv*. 2016:074161 https://doi.org/10.1101/074161
7. Pruesse E, Quast C, Knittel K *et al.* Silva: A comprehensive online resource for quality checked and aligned ribosomal rna sequence data compatible with arb. *Nucleic acids research*. 2007;**35**:7188-96
8. Caporaso JG, Kuczynski J, Stombaugh J *et al.* Qiime allows analysis of high-throughput community sequencing data. *Nature methods*. 2010;**7**:335-36
9. Rodriguez-r LM, Konstantinidis KT. Estimating coverage in metagenomic data sets and why it matters. *The ISME journal*. 2014;**8**:2349-51
10. Love MI, Huber W, Anders S. Moderated estimation of fold change and dispersion for rna-seq data with deseq2. *Genome Biol*. 2014;**15**:550 https://doi.org/10.1186/s13059-014-0550-8
